# Forewarned is forearmed: rice plants develop tolerance to post-anoxia during anoxic conditions by proteomic changes

**DOI:** 10.3389/fpls.2025.1647411

**Published:** 2025-09-30

**Authors:** Anton E. Shikov, Valeriya I. Shost, Tamara V. Chirkova, Maria F. Shishova, Vladislav V. Yemelyanov

**Affiliations:** ^1^ Faculty of Biology, St. Petersburg State University (SPbSU), St. Petersburg, Russia; ^2^ Laboratory for Proteomics of Supra‐Organismal Systems, All‐Russia Research Institute for Agricultural Microbiology (ARRIAM), St. Petersburg, Russia

**Keywords:** anoxia, re-aeration, proteomics, rice, stress tolerance, 2D-DIGE, transcription factors

## Abstract

**Introduction:**

In the absence of oxygen (anoxia), plants suffer from an energy shortage. Subsequent return to normoxia could exacerbate the obtained damage through severe oxidative stress. Thus, in nature, post-anoxia is a broad combination of stressors. The efficient recovery after oxygen depletion can occur only by the activation of defensive systems.

**Methods:**

In this study, we analyzed the impact of anoxia and re-aeration on tolerant rice at a proteomic level using two-dimensional gel electrophoresis followed by mass spectrometry. We further used bioinformatic predictions to reveal transcription factors modulating stress-induced gene expression.

**Results:**

Mass spectrometry revealed 82 spots corresponding to 13 and 8 unique proteins in shoots and roots, respectively. Spot-wise clustering illustrated that the re-aeration-related proteome resembles ones in the anoxic but not the control conditions. We classified proteins into four groups according to the intensities of spots under distinct conditions and observed that anoxia- and reoxygenation-specific proteins constituted a minor fraction (24%), unlike the other two. One of them contained proteins whose content continually decreased during stress, such as RuBisCO and fructose-bisphosphate aldolase. The second group included proteins whose synthesis started in anoxia and reached a peak during re-aeration. It involved OEE1 (oxygen-evolving enhancer protein 1), heat shock proteins, and pathogenesis-related (PR) proteins, implying defense from oxidative damage and pathogens to which plants become vulnerable during re-aeration. Promoter regions of genes encoding these proteins were enriched with transcription factor binding sites of stress-related TFs, both well-studied (ERF, WRKY, MYB) and not as frequently discussed in such contexts (TCP, TBP, SBP).

**Discussion:**

By comparing our observations with proteomic and transcriptomic research, we revealed that plant reactions to anoxia and reoxygenation are starkly similar. Extrapolating out results based on pure anoxia and reoxygenation, we suggest that rice shoots and roots become pre-adapted to the post-anoxic period in broad terms during oxygen depletion.

## 1 Introduction

Rice (*Oryza sativa*) is a food source for almost half of the world’s population (Sarkar et al., 2015). Due to the underwater growth, rice is tolerant to flooding and low oxygen stress (Mitsuya et al., 2009). Although rice possesses an arsenal of defense mechanisms, severe crop losses caused by flooding are reported annually (Paul and Rasid, 1993), especially with vulnerable cultivars (Hendrawan and Komori, 2021; Shrestha et al., 2021). The flooding-associated damage is predominantly caused by the low oxygen content (hypoxia) or complete absence of oxygen (anoxia) when the plant is submerged, i.e., located under the water (Jethva et al., 2022). Such conditions provoke severe energy depletion (Chirkova and Yemelyanov, 2018), production of toxic metabolites (Zuckermann et al., 1997; Yemelyanov et al., 2023), cytoplasmic acidification (Kulichikhin et al., 2009), generation of reactive oxygen species (ROS) (Blokhina et al., 2001; Shikov et al., 2021a), and oxidation of cellular components (Chirkova et al., 1998; Shikov et al., 2021a).

Adaptive strategies to combat oxygen deficiency represent a continuum with two extremes termed low-oxygen escape syndrome (LOES) and low-oxygen quiescence syndrome (LOQS) (Voesenek and Bailey-Serres, 2013; Shikov et al., 2020). The first implies morphological changes, namely, rapid shoot growth (Bailey-Serres and Voesenek, 2008), aerenchyma formation (Mignolli et al., 2020), and adventitious root development (Rich et al., 2008). LOES is governed by phytohormones, e.g., ethylene (Sauter and Steffens, 2014; Hu et al., 2019), auxin (Eysholdt-Derzsó and Sauter, 2017), and gibberellins (Ayano et al., 2014), coupled with suppression of abscisic acid signaling (Yemelyanov and Shishova, 2012). Other mediators include transcription factors SNORKEL1 (SK1) and SNORKEL2 (SK2) belonging to the ERF-VII (ethylene response factor) family (Hattori et al., 2009), SnRK1A (sucrose-nonfermenting1-related protein kinase 1A), and CIPK15 (calcineurin B-like protein-interacting protein kinase 15) kinases (Lee et al., 2009). Contrarily, the quiescence strategy induces growth retardation (Luo et al., 2012) and the concomitant metabolic adjustments (Jethva et al., 2022). The latter represent intensive energy conservation (Voesenek and Bailey-Serres, 2015), maintenance of membrane transport (Bailey-Serres and Voesenek, 2010), switch to glycolysis and fermentation (Fukao et al., 2019), as well as protection from oxidative stress by antioxidants (Blokhina et al., 2000) and heat shock proteins (Banti et al., 2010). LOQS is triggered by SUB1A (Submergence 1A), the ERF-VII family transcription factor, which suppresses gibberellin and ethylene biosynthesis (Schmitz et al., 2013) and inhibits sucrose synthase (Fukao et al., 2006).

While the impact of low oxygen on plants is relatively well researched, our understanding of plant responses to post-hypoxic and post-anoxic exposure remains limited. Nevertheless, evidence shows that severe oxidative stress observed during re-aeration (Shikov et al., 2020, 2022; Jethva et al., 2022) is associated with the generation of ROS (Blokhina et al., 2000), lipid peroxidation (Garnczarska and Bednarski, 2004), destruction of membranes (Pavelic et al., 2000), accumulation of acetaldehyde (Luo et al., 2012) and methylglyoxal (Devanathan et al., 2014), reduction in biomass (Li et al., 2021), chlorophyll degradation (Hemschemeier, 2013), and programmed cell death (Tamang et al., 2014). After anoxia, plants suffer from desiccation due to the impaired osmotic regulation (Setter et al., 2010) and become susceptible to pathogens (Tamang and Fukao, 2015). Despite tolerance to oxygen depletion per se, certain cultivars of *Oryza glaberrima* are vulnerable to oxidative stress during re-aeration (Steudel et al., 2009; Sakagami et al., 2013).

To reduce ambiguity, in this research, we will distinguish the terms re-aeration and post-anoxia, while in the literature, they are frequently used as synonyms. Re-aeration (or reoxygenation) is defined as pure return to oxygen-containing conditions after anoxic treatment, while post-anoxia is perceived as a more general term related to the natural occurrences of re-aeration as a consequence of distinct events, e.g., flooding, waterlogging, and other phenomena in nature. Thus, when describing post-anoxia, we imply extrapolation of our findings, possibly reflecting rice adaptations to the natural post-anoxic exposure caused by various factors.

Therefore, post-anoxia per se is a combination of stress conditions, including direct consequences of anoxic damage, oxidative stress, and desiccation (Shikov et al., 2020). For this reason, resistance to post-anoxia involves multiple defensive mechanisms. First, toxic acetaldehyde is metabolized by aldehyde dehydrogenase (ALDH), pyruvate dehydrogenase complex (PDC) (Tsuji et al., 2003), and betaine aldehyde dehydrogenase (Mitsuya et al., 2009) for rapid ATP production (Mustroph et al., 2006). Methylation and acetylation of H3 histone in *ADH1* and *PDC1* genes in rice during anoxia enable increased gene expression when returning to an oxygen-containing environment (Tsuji et al., 2006). Methylglyoxal is detoxified by lactoglutathione lyase (GLX1) and hydroxyacylglutathione hydrolase (GLX2) (Devanathan et al., 2014). Second, the energy state is restored by metabolism of γ-aminobutyrate, lactate, and alanine accumulated during hypoxia/anoxia with aminotransferase/glutamate dehydrogenase (Mustroph et al., 2014) and enzymes of the tricarboxylic acid cycle (TCA) (Shingaki-Wells et al., 2014; Yemelyanov et al., 2023). Additional energy could be generated by the consumption of stored carbohydrates, e.g., fructans (Albrecht et al., 1993). Third, plants combat oxidative stress through the synthesis of antioxidants, namely, glutathione (Biemelt et al., 1996), ascorbic acid (Ushimaru et al., 1992), tocopherols (Blokhina et al., 2000), and the activity of antioxidant enzymes, including superoxide dismutase (SOD) (Monk et al., 1989), catalase, and peroxidase (Monk et al., 1987; Garnczarska and Bednarski, 2004; Yemelyanov et al., 2022), as well as the components of the ascorbate-glutathione cycle (Biemelt et al., 1998; Yemelyanov et al., 2024). Antioxidants and enzymes jointly prevent lipid damage (Chirkova et al., 1998) and maintain the stability of PSII (Barik et al., 2019). Finally, late embryogenesis abundant (LEA) proteins, osmolyte biosynthesis enzymes, and chaperones contribute to overcoming desiccation (Fukao et al., 2011). Re-aeration-induced gene expression is regulated by phytohormones, including ethylene (Tsai et al., 2014), jasmonic acid (Yuan et al., 2017), and ABA (Jethva et al., 2022), as well as stress transcription factors, ERF1/ERF2 (Tsai et al., 2014), EREBP1 (Tamang and Fukao, 2015), SUB1A (Alpuerto et al., 2016), and WRKY (Raineri et al., 2015). It is worth mentioning that different organs of the same plant are unequally affected by re-aeration. For instance, in wheat and soybeans, lipid peroxidation was more pronounced in shoots (Da-Silva and Amarante, 2020; Shikov et al., 2022). On the contrary, in barley and *Arabidopsis thaliana* plants, shoots were less damaged by reoxygenation than roots (Ellis et al., 1999; Skutnik and Rychter, 2009).

Therefore, developing new strategies to lower flooding-induced crop loss requires studying the impact of oxygen depletion and re-aeration on plants. This necessity is emphasized by steadily increased flooding caused by global climate change (Tamang and Fukao, 2015; Voesenek and Bailey-Serres, 2015; León et al., 2021). Proteomic approaches are comprehensive methods illustrating translational changes in plants exposed to stress conditions. In rice, two-dimensional proteomics revealed responses to wounding (Shen et al., 2003), cold stress (Cui et al., 2005), bacterial blight (Mahmood et al., 2006), blast fungus (Kim et al., 2009), drought (Liu and Bennett, 2011), salinity (Song et al., 2011), high temperature (Kumar et al., 2017), etc. However, to the best of our knowledge, only a few proteomes of plants subjected to anoxia followed by re-aeration have been studied so far, e.g., the mitochondrial proteome of rice seedlings (Millar et al., 2004) and the roots of soybean plants (Salavati et al., 2012). To fill the gap, we conducted a two-dimensional difference protein gel electrophoresis (2D-DIGE) study to reveal the effect of 24 h of pure anoxia followed by 24 h of re-aeration on rice shoots and roots. By applying several clustering approaches, we show that the proteomic landscapes of plants exposed to anoxia and re-aeration group together, implying similar stress effects. We reported proteins specific to re-aeration, such as oxygen-evolving enhancer proteins, heat shock proteins, and chitinases. Finally, we analyzed promoter regions of genes encoding these proteins and found possible transcription regulators belonging to stress-related TFs, such as widely reported WRKY, Myb/SANT, and ERF, coupled with less-studied families in the context of anoxia/re-aeration, namely, TCP, SBP, and TBP.

## 2 Materials and methods

**Figure 1:**
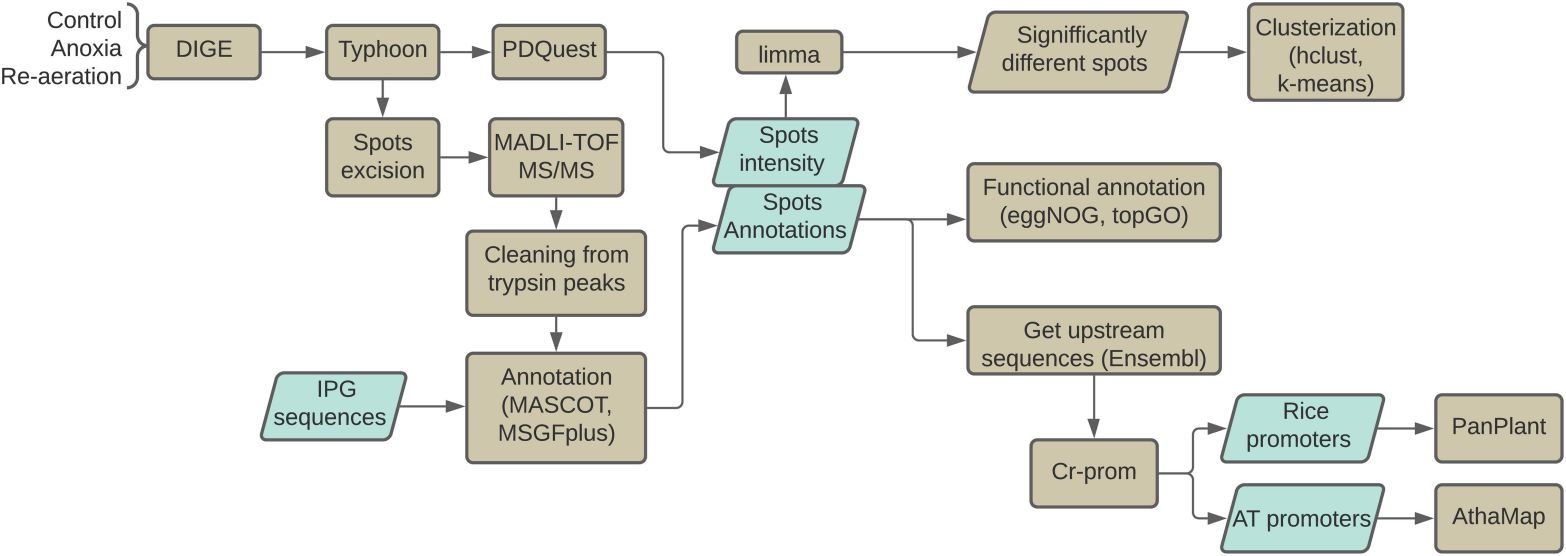
The overview of the study. Plants were exposed to 24 h of pure anoxia followed by 24 h of re-aeration. 2-DIGE gel figures were scanned with Typhoon, and the intensities of spots were calculated using the PDQuest software. After excision, spots were analyzed with MALDI-TOF MS/MS. Proteins were identified using MASCOT and MGSFplus utilities. Significantly different spots were identified with the limma package. Individual spots and the whole proteomes were then clustered with k-means and hierarchical clustering methods. Functional annotation of protein sequences was performed using the eggNOG standalone tool v2.0.1b-2-g816e190 (Huerta-Cepas et al., 2017) and the topGO v.2.34.0 (Alexa and Rahnenfuhrer, 2007) package. The upstream regions of genes encoding identified proteins and the respective orthologs from *A*. *thaliana* were obtained from the EnsemblPlants (Bolser et al., 2016) database. Finally, transcription factor binding sites within the promoter regions were predicted using the PlantPAN v.3.0 (Chow et al., 2019) and AthaMap (Hehl et al., 2016) tools.

The proteomic study described here was conducted according to the following scheme (**Figure 1**). All scripts, as well as the raw data for statistical comparisons described in the study, are available in the Git repository (https://github.com/anton-shikov/Rice_proteomics).

### 2.1 Plant material

Ten-day-old rice seedlings (*Oryza sativa* L., cv. Flagman, Federal Scientific Center of Rice, Belozerny, Krasnodar, Russia) were studied. Seeds were sterilized using 5% NaClO for 15 min and washed with warm distilled water. Seeds were then soaked for 1 h in hot water (50-55°С) to induce germination in dark conditions for 3 days at 28°С. Germinated seedlings were planted on perforated plastic plates on containers filled with continuously aerated Knop nutrient solution (0.2 strength) and grown at an irradiance of 60 µmol * m^-2^ * s ^-1^ with a photoperiod of 12 h at 23-25°C as described earlier (Emel’yanov et al., 2003; Yemelyanov et al., 2020).

### 2.2 Experimental setup for modelling anoxia and re-aeration

Plants were divided into control and experimental groups. For each condition (control, anoxia, and re-aeration), 3 glass beakers containing 20 mL of Knop nutrient solution (0.2 N strength) with 20 seedlings in each were used. To replicate anaerobic conditions, beakers with seedlings were placed into 1.5-liter jars (exicators). The gaseous nitrogen with less than 0.01% of oxygen was pumped to the exicators for 45 min until reaching complete anoxia. The purity of anaerobic conditions was confirmed via the Anaerotest^®^ anaerobic indicator (Merck, Darmstadt, Germany). Once anaerobic conditions were reached, exicators were tightly closed and put in the dark for 24 h to prevent oxygen production during photosynthesis. Control plants were exposed to the dark in aerobic conditions for 24 h. Three biological replicates were inspected for each condition. After 24 h of anoxia, the jars were opened, followed by collecting plant shoots and roots for further protein extraction in the case of anoxic proteomes and control settings. The beakers with anoxia-treated plants were further exposed to 24 h of dark aerobic conditions (re-aeration) and then were separately fixed for protein extraction as well. Dark conditions during the reoxygenation state were retained to prevent from possible additional generation of ROS caused by the effect of light on the damaged photosynthetic apparatus to analyze the role of re-aeration per se.

### 2.3 Protein extraction

To extract proteins, we modified the methodology from the proteomic studies (Levine et al., 1994; Komatsu et al., 2014). Seedlings (2 g of shoots and 4 g of roots) were homogenized with liquid nitrogen using a mortar and pestle in a 1.5 ml TE extraction buffer (50 mM Tris-HCl, 5 mM EDTA, pH 7.5), followed by adding 5 μl of protease inhibitor cocktail (Sigma-Aldrich, Saint Louis, MO, USA). After 15 min of extraction under +4°С, samples were centrifuged for 10 min at 15000 g, +4°С. Protein concentration was assessed by micro-volume Bradford assay with the Spekol 1300 spectrophotometer (Analytik Jena, Jena, Germany). The volumes of the samples were calculated to obtain 120 μg of total protein. The respective solutions were mixed with 40% TCA in a 1:1 ratio, vortexed, and kept on ice for 10 min to form a protein pellet. Subsequently, samples were centrifuged for 10 min at 15000 g, +4°С. The pellets were then resuspended in 1 ml of washing liquid I (10% TCA, 0.07% β-mercaptoethanol in pure acetone) for 1 h in the freezer at -20°С. Next, samples were centrifuged again for 10 min at 15000 g, +4°С and resuspended in 1 ml of washing liquid II (0.07% β-mercaptoethanol in pure acetone) for 10 min and centrifuged under the same conditions. The procedure using washing liquid II was repeated twice. The samples were then dried with SpeedVac (Thermo Fisher Scientific, Waltham, MA, USA) at 10°С в for 10 min. The protein pellet was diluted in lysis buffer (7 M Urea, 2 M Thiourea, 4% CHAPS, 25 mM Tris, pH 8.2, 50 mM DTT) to obtain 40 μg of proteins per sample by sonication on ice for 20 min using the Elmasonic S 10H sonicator (Elma Electronic, Wetzikon, Switzerland) and vortexing for 5 min.

### 2.4 Two-dimensional fluorescent difference gel electrophoresis

To mark condition-specific proteins, the samples were conjugated with Cy2, Cy3, or Cy5 dyes (Lumiprobe, Hunt Valley, MD, USA) in a proportion of 400 pM of a dye to 50 µg of total protein (0.9 µl per 30 µM of the sample). After adding dyes, samples were vortexed and conjugated on ice in the dark for 15 min. To terminate conjugation, the incubation with 1 µl of 10 µM *L*-lysin for 15 min on ice was applied. When termination was completed, samples were mixed in equal volumes, and 30 µl of biolytes were added (50 мМ DTT, 1% Biolite, Bio-Rad Laboratories, Hercules, CA, USA). The resulting mixture with samples from all conditions (125 µl) was loaded into the IPG strip (7 cm, pH 3–10; Bio-Rad Laboratories, Hercules, CA, USA). Strip loading was performed by overnight rehydration at room temperature, and with 1 ml of mineral oil (Bio-Rad Laboratories, Hercules, CA, USA) added above the strip to prevent drying of the strip during the process. When the rehydration process was finished (12 h), strips were cleaned from mineral oil and placed into PROTEAN^®^ i12™ IEF (Isoelectric Focusing) System (Bio-Rad Laboratories, Hercules, CA, USA) with Electrode Wicks wetted by 8 µl of deionized water. Manufacturer-recommended parameters were used for IEF (10,000 V/h, end voltage 4000 V, maximal current 50 mA per IPG-strip, rapid voltage ramp, 20°C). Next, IEF-subjected strips were incubated in 1 ml of equilibration buffer I (6 M urea, 2% SDS, 0.375 М Tris, 20% glycerin, 2% dithiothreitol, pH 8.8) for 15 min and 1 ml of equilibration buffer II (with 2.5% iodoacetamide instead of dithiothreitol and 20 µl of 0.5% bromophenol blue solution). The second direction, i.e., SDS-PAGE (Laemmli, 1970), was performed in a 4% concentrating gel and 15% separating gel in Mini-PROTEAN^®^ Tetra Cell Systems (Bio-Rad Laboratories, Hercules, CA, USA). Ten μl of PageRuler™ protein ladder (Thermo Fisher Scientific, Waltham, MA, USA) was added. Electrophoresis was performed in Tris-glycine buffer (25 mM Tris, 192 mM glycine, 0.1% SDS, pH 8.5) at 200 V. Different Cy-dyes were visualized using the Typhoon FLA 9500 laser scanner with default settings (GE Healthcare, Chicago, IL, USA). The intensities of the protein-containing spots were calculated using the PDQuest v.8.0 (Bio-Rad Laboratories, Hercules, CA, USA) utility. Gels of different plant organs were then analyzed separately.

### 2.5 Protein mass spectrometry

To mark spots, gels were stained with Coomassie brilliant blue G-250 for 1 h. Spots of interest chosen according to the PDQuest results. The respective regions were excised from gels and cut into small pieces approximately 1 mm^2^-sized following the “bottom-up” approach described earlier (Maltseva et al., 2020; Shikov et al., 2021b). The stain was removed by incubation with 50 mM NH_4_HCO_3_ in 50% acetonitrile for 15 min. After that, samples free from the buffer were incubated in 100% acetonitrile for 5 min. The procedure was repeated twice. Samples were dried using the centrifugal vacuum concentrator CentriVap (Labconco, Kansas City, MO, USA) at 18°C. Dried samples were rehydrated with trypsin solution (20 ng/µl, 25 mM NH_4_HCO_3_, pH 8.2, Sigma-Aldrich, Saint Louis, MO, USA) on ice for 60 min. After trypsin removal, 30 µl of 25 mM Tris (pH 8.2) was added to each sample. Trypsinolysis lasted for 12–18 h (overnight) at 37°C. Digested peptides were extracted in two steps by adding 20 μl of 25% and 50% acetonitrile and collecting the supernatant. Samples were then dried using the centrifugal vacuum concentrator CentriVap at 18°C. The samples were scanned in the positive ion detection mode after ionization with the matrix-activated laser (MALDI) approach. The matrix was composed of α-cyano-4-hydroxycinnamic acid (Bruker Daltonics, Bremen, Germany). MS fingerprints and MS/MS spectra were obtained with the MALDI-TOF/TOF mass spectrometer Ultraflextreme (Bruker Daltonics, Bremen, Germany) in the reflector positive tandem (TOF/TOF ion mode with a stainless-steel target and in a mass range from 700 to 3500 Da. The internal calibration mass measurement error was no more than 1.5 ppm, while the external did not exceed 5 ppm. The adjustment of raw spectra was conducted with the Bruker Flex software (Bruker, Billerica, MA, USA).

Raw files in the Bruker flex XML format were transformed into tab-delimited text files for peptide fingerprinting MS1 spectra and MGF (Mascot Generic Format) files for MS/MS spectra using a custom Bash script. MGSF files were processed with a custom Python 3.7 script to remove peaks corresponding to trypsin autoproteolysis m/z estimates. The respective values were predicted using *in silico* proteolysis of the reference trypsin sequence (UniProt accession – P00760) with PeptideMass from the ExPASy server (Gasteiger et al., 2003). Protein identification was performed using the UniProtKB/SwissProt database (June 2021, 565254 sequences) and Identical Protein Groups (https://www.ncbi.nlm.nih.gov/ipg/, June 2021, 204587 sequences of *O*. *sativa*). MS/MS-based identification was carried out with two tools, namely, the Mascot server (Matrix Science, London, UK) and the MSGFplus v.3.12 package (Kim and Pevzner, 2014) for R 4.1.2. The following parameters were specified: (i) peptide tolerance 0.6 Da (0.8 Da for MS1 spectra), (ii) MS/MS tolerance 0.2 Da, (iii) peptide charge 1+, (iv) monoisotopic mass, (v) trypsin cleavage with up to 2 missed cleavages, (vi) fixed cysteine carbamidomethylation and variable methionine oxidation modifications, (vii) score > 40 (>9 for MSGFplus annotations).

When using MSGFplus, the ‘runMSGF’ function was applied to MGF files. Additionally, the MsnID v.1.29.0 package was applied to filter the obtained results. The respective post-processing included the following procedures: (i) check of the peptide termini conformance with cleavage (‘assess_termini’ function), (ii) counting missing cleavage sites (‘assess_missed_cleavages’ function), (iii) log10-transforming spectrum E-value, (iv) filtering spectra with ‘MSnIDFilter’, and (v) optimizing filter with Nelder-Mead optimization (Piehowski et al., 2013). In both approaches, a decoy search mode was specified, and only significant non-random hits were reported (p-value ≤ 0.05). Protein annotations were retained if at least one identification (MS1 and/or MS/MS) was successful. The priority was given to MS/MS spectra-based results. The reference protein sequences were downloaded from the NCBI Protein database (https://www.ncbi.nlm.nih.gov/protein). All the determined annotations were combined and checked for duplicates with the CD-HIT v.4.8.1 utility with a 100% clustering threshold and a word size of five letters (Fu et al., 2012). The same accession numbers were assigned for duplicates. The general properties of the proteins were predicted with the «compute pI/Mw» tool from the ExPASy server (Gasteiger et al., 2003). Molecular weight and isoelectric points of the spots were calculated with ImageJ v.1.46r by identifying the Rf values on raw gel figures.

### 2.6 Statistical analysis of the spots from proteomes under distinct conditions

The data was visualized with the ggplot2 package v3.3.2 (Wickham, 2016). The per-gels PDQuest tables were merged into tables with a custom R script. Data were log_2_-transformed, averaged, and normalized with the ‘normalizeQuantiles’ function from the limma package v.3.28.14 (Ritchie et al., 2015). Several statistical comparisons were then performed between organs and conditions using the t-test with p-values adjusted with FDR (False Discovery Rate) correction for multiple comparisons. To find significantly different spots, three statistical tests were employed, namely, (i) the Kruskal-Wallis test with the ‘kruskal.test’ function, (ii) a mixed linear model with samples specified as a random variable using the ‘lme’ function from the nlme package v.3.1-152 (Pinheiro et al., 2012), and (iii) a linear model with Bayes statistics evaluation applying the ‘lmFit’ and ‘eBayes’ utilities from limma (Ritchie et al., 2015). Three methods for p-value adjustments were applied as well: (i) FDR, (ii) Holm (Holm–Bonferroni), and (iii) BH (Benjamini-Hochberg). Spots were then divided into four groups for further analysis: (i) all spots, (ii) significantly different spots, (iii) all spots with known annotations, and (iv) significantly different spots possessing annotations. In this research, we simply call these groups (i) all, (ii) significant, (iii) annotated, and (iv) significant annotated spots. Heatmaps with normalized averaged intensities for each group were generated with the ‘Heatmap’ function from the ComplexHeatmap package v.2.9.3 (Gu et al., 2016).

### 2.7 Clustering the proteomic whole inferences of samples and individual spots

Next, we used two clustering algorithms, namely, k-means and hierarchical clustering, to reveal groups of spots and samples with similar patterns of condition-wise intensities. The optimal number of clusters for the k-means procedure was evaluated with the elbow method (Demidenko, 2018) by manual inspection of the with-in-Sum-of-Squares (WSS) estimates. Clustering results were visualized with the ‘autoplot’ function from the ggfortify v0.4.11 (Tang et al., 2016) package. The complete method was specified to perform hierarchical clustering. The optimal number of clusters was chosen according to the maximal silhouette score evaluated with the ‘silhouette’ function from the cluster v.2.1.2 (Maechler et al., 2021) package. Mean intensities of the spots under distinct conditions were assessed with a custom Python script. The clustering protocols were compared with two similarity assessment methods. Within a single clustering approach, we calculated the sum of maximal Simpson coefficients (the intersection of two sets per the minimal power of two sets) between clusters divided by the maximum number of clusters, as in Equation 1. When comparing different clustering procedures within the same type, the Jaccard coefficient (the intersection of two sets per the union of the sets) was applied, as in Equation 2:


(1) $$\text{S}=\frac{\sum_{\text{i},\max(|\text{K}|,|\text{M}|)}\frac{\left|{\text{K}}_{\text{i}}\cap {\text{M}}_{\text{i}}\right|}{\min(|{\text{K}}_{\text{i}}|,|{\text{M}}_{\text{i}}|)}}{\max(|\text{K}|,|\text{M}|)}$$

(2) $$\text{S}=\frac{\sum_{\text{i},\max(|\text{K}|,|\text{M}|)}\frac{\left|{\text{K}}_{\text{i}}\cap {\text{M}}_{\text{i}}\right|}{\left|{\text{K}}_{\text{i}}\cup {\text{M}}_{\text{i}}\right|}}{\max(|\text{K}|,|\text{M}|)}$$

where S is an integral similarity, K is a set of the first clustering group, M is a set of the second clustering group, |K| and |M| denote the number of clusters in each set, and K_i_ and M_i_ are the subsets of spots within the i-th cluster in the respective clustering groups, |K_i_| and |M_i_| stands for the number of spots for the i-th cluster in the clustering group, finally |K_i_ ∩ M_i_| and |K_i_ ∪ M_i_| denotes the number of common spots the total number of spots for i-th clusters.

### 2.8 Functional annotation

Deduplicated sequences were functionally annotated using the eggNOG standalone tool v2.0.1b-2-g816e190 (Huerta-Cepas et al., 2017) in the MMSeqs2 (Steinegger and Söding, 2017) search mode. The respective COG (Cluster of Orthologous Genes) attributions were summarized for each organ separately. To obtain the GO (Gene Ontology) enrichments, we applied the over-representation test with the topGO v.2.34.0 (Alexa and Rahnenfuhrer, 2007) package using Fisher’s statistics and the FDR p-value adjustment procedure. The universe of GO terms derived from protein sequences for both organs.

### 2.9 Upstream *cis*-elements analysis

To get promoter regions, protein sequences were used in the BLAST search in the EnsemblPlants (Bolser et al., 2016) database against the latest reference genomes of *O*. *sativa* and *A*. *thaliana*. The upstream elements of the *A*. *thaliana* plants were chosen for comparative purposes, since Arabidopsis is more vulnerable to anoxic conditions compared with rice (Narsai and Whelan, 2013). The 500 bp upstream regions to the respective genes in the rice genome were then downloaded, while for *A. thaliana*, gene identifiers were saved. The sequences were assessed as true promoters with the CrProm software (Shujaat et al., 2021) by cutting the regions into two 250 bp-long sequences and processing them with the Cr-Prom web interface. Only the past promoters remained for further analysis. Transcription factor binding sites (TFBS) were then predicted with the PlantPAN v.3.0 utility (Chow et al., 2019) and the AthaMap web server (Hehl et al., 2016) in the case of Arabidopsis. The total number of sites, their location, and the frequency of the particular TF family were summarized with a custom Python script.

## 3 Results

### 3.1 Protein identification results for shoot and root proteomes under anoxia and re-aeration

**Figure 2:**
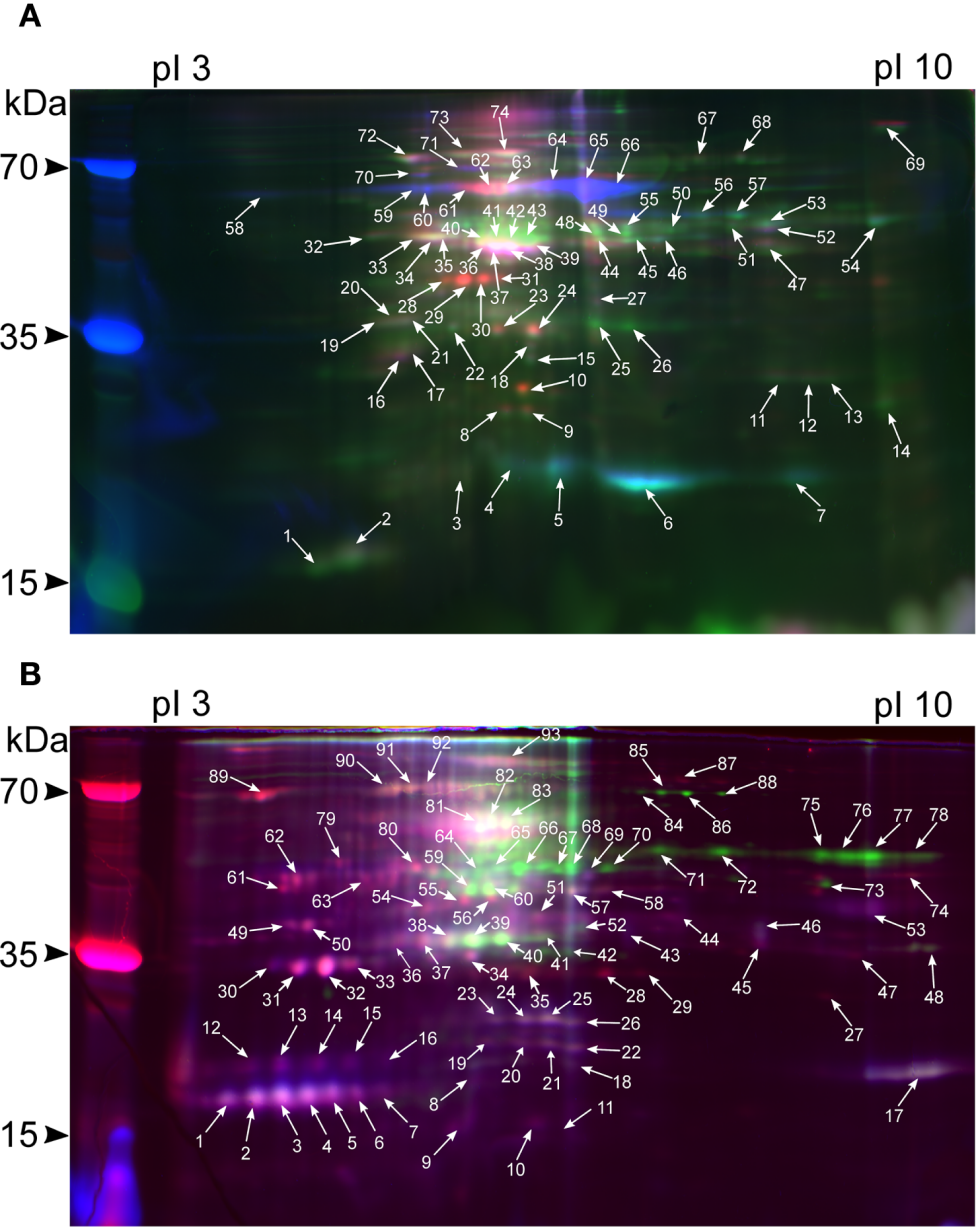
2D-DIGE image corresponding to the overlapping Cy2, Cy3, and Cy5 fluorochrome channels of rice shoot **(A)** and root **(B)** proteomes during different experimental conditions. The green channel indicates control, normoxia, blue – anoxia, and red – re-aeration. The proteins detected by MS/MS are presented in **Supplementary Tables 2, 3**.

**Figure 3:**
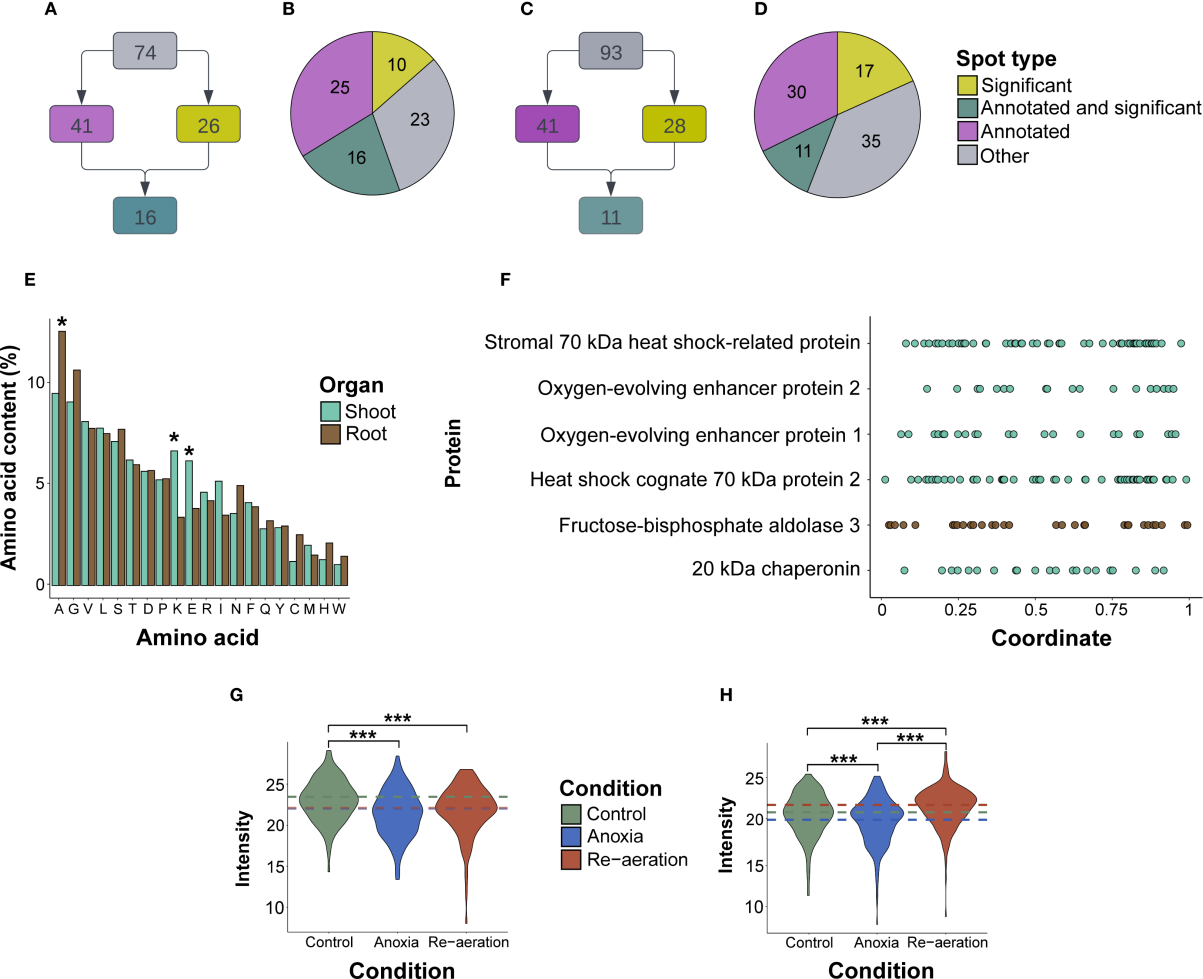
General characteristics of excised spots and the respective proteins. **(A)** The scheme of classifying spots in shoots and roots **(B)** regarding the level of significance and the presence of MS/MS annotations. **(C)** Distribution of spots in shoot and root **(D)** proteomes corresponding to four groups (significantly different spots, significantly different spots with protein annotations identified by mass spectrometry, all spots with annotations, and the remaining ones). **(E)** The mean percentage of amino acid residues in shoot and root proteins. Asterisks denote significant differences in a certain residue percentage between shoot and root proteins. **(F)**. Coordinate-wise distribution of lysine residues according to the relative positions within protein sequences. The underlying data used for generating the two former plots are given in **Supplementary Tables 5, 6**. The average intensities of protein spots in shoot **(G)** and root **(H)** proteins under control and experimental conditions. The intensity was calculated by the PDQuest utility. The colored dashed lines represent the overall gel-wise median values. The samples were compared using a pair-wise t-test adjusted for multiple comparisons using the FDR (False Discovery Rate) method. Asterisks denote significant differences according to the Wilcox test with p-values adjusted using the Benjamini-Hochberg procedure (* — p < 0.05, ** — p < 0.01, *** — p < 0.001).

A total of 167 (74 for shoots and 93 for roots) spots were excised from gels (**Figures 2A, B**). A total of 101 meaningful MS1 and 151 MS/MS spectra were obtained (**Supplementary Table 1**). In shoots, 41 spots were identified (**Figures 3A, B**), four of which possessed MS1 spectra only, 14 – MS/MS spectra only, and the other 22 had both MS/MS and MS1 (**Supplementary Table 2**). In roots, of 41 spots in total (**Figures 3C, D**), one spot had MS1 spectra only, 28 – MS/MS only, and 11 possessed both, respectively (**Supplementary Table 3**).

Predicted and experimentally observed isoelectric points and molecular masses of identified proteins were highly congruent according to the Pearson (p-value< 1.697e-07) (**Supplementary Figures 1A, B**; **Supplementary Table 4**). Root proteins were significantly more acidic (pI 6.08 vs. pI 6.65) and with a lower molecular weight (34 kDa vs 45 kDa) than shoot proteins (p-value< 0.001 according to the t-test). On average, shoot proteins were significantly enriched with positively and negatively charged lysine and glutamic acid, while root proteins contained more alanine (p<0.01 according to the t-test), which could explain pI differences (**Figure 3E**; **Supplementary Table 5**). We also analyzed the percentage of lysine residues. Most of the proteins contained two to three regions enriched with lysine (**Figure 3F**). In shoots, lysine-rich proteins were identified in the spots that accumulated during re-aeration, namely, HSP70 and oxygen-evolving complex proteins (**Supplementary Table 6**). Conversely, in roots, proteins contained two times fewer lysine residues, except for cytoplasmic fructose-bisphosphate aldolase 3 (**Supplementary Table 6**).

### 3.2 Statistical differences between the protein spots under distinct conditions

According to the log_2_-transformed and normalized (**Supplementary Figures 2A-D**; **Supplementary Table 7**) intensities of the protein spots, in shoots, the mean intensity during re-aeration was equal to anoxic conditions and decreased relative to normoxia (**Figure 3G**). In roots, mean intensities of protein spots during re-aeration exceeded the respective estimates in anoxia and control, indicating possible re-aeration-specific protein production (**Figure 3H**).

We applied several statistical frameworks to obtain significantly different spots (**Supplementary Table 8**) and selected the Bayesian test as the most optimal. The approach detected 26 significantly different spots in shoots, 16 of which were attributed to protein annotations (**Supplementary Table 9**). The amount of the small subunit of ribulose-1,5-bisphosphate carboxylase/oxygenase, fructose-bisphosphate aldolase, phosphoribulokinase, and sedoheptulose-1,7-bisphosphatase gradually declined from control to re-aeration. The intensity of spots corresponding to the large subunit of RuBisCO and heat shock cognate 70 kDa protein 2 reached a peak in anoxic conditions, with a subsequent decrease during reoxygenation. Other proteins, namely, 20 kDa chaperonin, chloroplastic isoform X1, oxygen-evolving complex protein 1 and 2, photosynthetic NDH subunit of lumenal location 5, chloroplastic stromal 70 kDa heat shock-related protein, and transketolase were gradually elevated from control to re-aeration. Finally, chloroplastic 2-Cys peroxiredoxin BAS1, after a decrease in anoxia, reached a peak during re-aeration.

In roots, 28 significantly different spots were identified, with 11 of them corresponding to mass spectrometric annotations (**Supplementary Table 10**). Cytoplasmic fructose-bisphosphate aldolase 3 and peroxidase P7 steadily decreased, reaching a minimum during re-aeration. The amount of the Prb1 protein increased during anoxia and declined after 24 h of re-aeration. Acidic PR-1 type pathogenesis-related protein, glucan *endo*-1,3-β-glucosidase 3 isoform X2, Cysteine-rich receptor-like protein kinase 6, and chitinase 2 showed a considerable accumulation in anoxia and re-aeration. Finally, the spots containing the alpha-*L*-arabinofuranosidase 1 isoform X3 restored their intensity during re-aeration after the anoxic decline.

### 3.3 Clustering patterns of samples and individual spots

**Figure 4:**
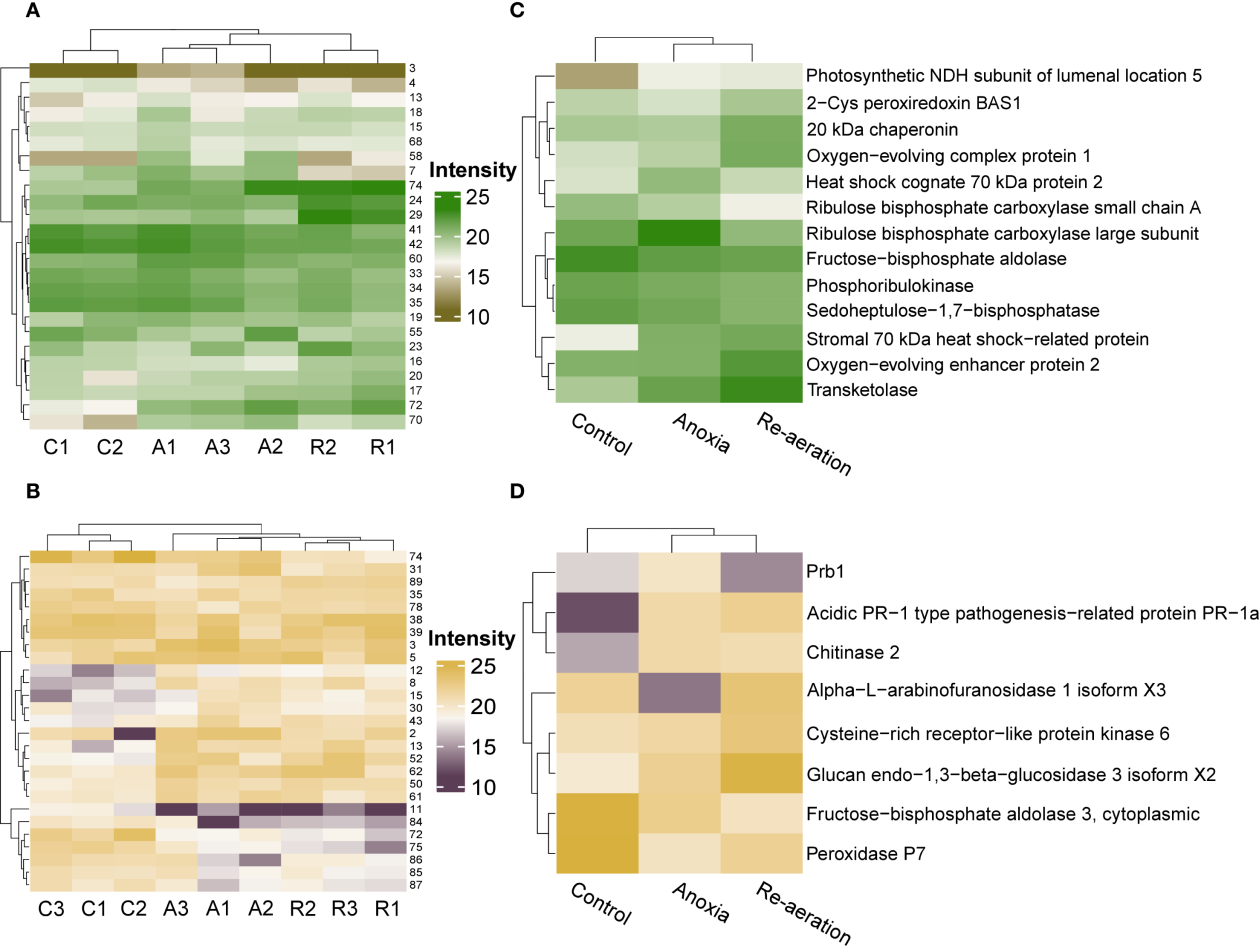
Heatmaps of the shoot **(A)** and root **(B)** proteomes with respective hierarchical clustering of samples based on significantly different protein spots. Experimental conditions are encoded by capital letters as follows: C – control, A – anoxia, and R – re-aeration. The right most adjacent numbers correspond to spots in **Figures 2A, B**. Heatmaps of shoot **(C)** and root **(D)** proteins identified with mass spectrometry, displaying the mean intensities in different spots harboring them. In all figures, the relative intensity was evaluated with the PDQuest software. To see the exact intensities, consult **Supplementary Tables 9-10**.

To characterize the patterns of proteomic changes under anoxia and re-aeration, we applied the k-means and hierarchical clustering procedure based on the four groups of spots as mentioned earlier (**Figures 2A–D**). In shoots, the k-means-based clustering results showed slight incongruence. The samples attributed to re-aeration and control formed a single cluster apart from the anoxic samples (**Supplementary Figures 3A–H**). However, hierarchical clustering revealed that re-aeration and anoxia proteomes tend to fall into one cluster (**Figure 4A**; **Supplementary Figures 4A–D**; **Supplementary Table 11**). In roots, both k-means (**Supplementary Figures 5A–H**; **Supplementary Table 11**) and hierarchical clustering (**Figure 4B**; **Supplementary Figures 6A–D**; **Supplementary Table 11**) demonstrated that anoxia is grouped with re-aeration in terms of spot-wise protein levels.

We then carried out clustering of individual spots of the four groups described earlier (see section 2.6) using two algorithms. First, we analyzed how the clusters correspond to each other by calculating the mean Simpson coefficient to find if the smaller cluster is a subset of the bigger (see section 2.7). When comparing k-means clusters, we found that most of the smaller groups were embedded into the larger (**Supplementary Figures 7A, B**). However, according to the hierarchical clustering procedure, most of the groups were distinct from each other (**Supplementary Figures 7C, D**). Second, we compared two clustering algorithms within the group of spots using the Jaccard coefficient, displaying the similarity between spot-wise clustering patterns. We found that the k-means clusters diverged substantially from hierarchical clusters for most of the groups (**Supplementary Figure E**). Moreover, k-means clustering provided 2 clusters for each group (**Supplementary Figures 8A–H**, **9A–H**; **Supplementary Table 12**), while hierarchical clustering resulted in 2 to 5 clusters (**Supplementary Table 12**).

**Figure 5:**
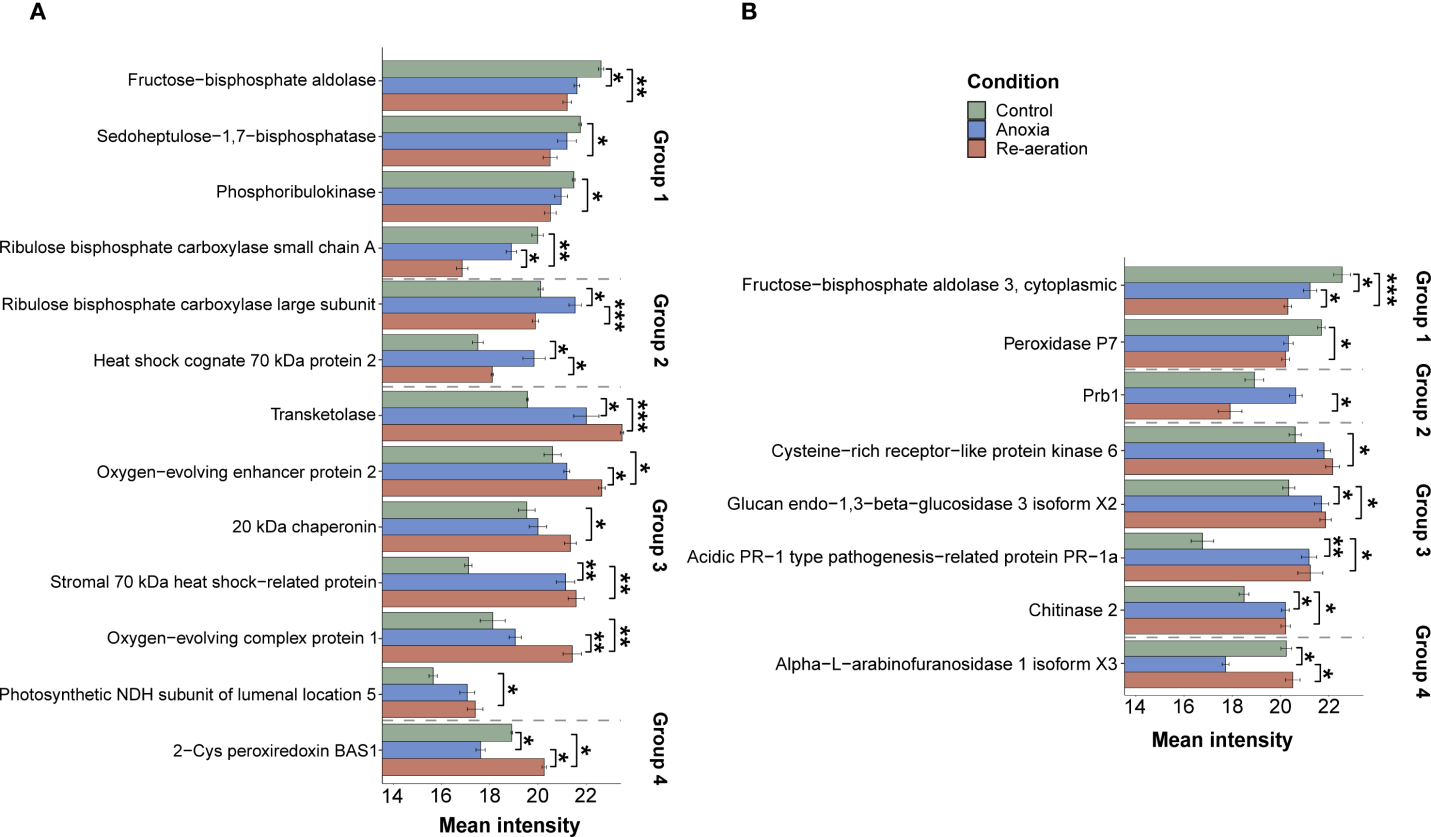
Mean intensities of significantly different and annotated protein spots of shoots **(A)** and roots **(B)** under control and experimental conditions. The error bars represent the standard error of the mean. According to the changes in abundance in stress conditions, proteins are categorized into four groups. The first group is represented by the proteins that slightly or drastically decreased in their amount during anoxic exposure and re-aeration. Proteins attributed to the second group of spots reach a peak during anoxia, with a subsequent dramatic drop after 24 h of re-aeration. The third group of proteins includes those that started to increase during anoxia and reached a peak in reoxygenation conditions. Proteins from the fourth group decreased during anoxia and sufficiently accumulated after 24 h of reoxygenation. The relative intensity of spots with the proteins was calculated using PDQuest. Statistical comparisons between the intensity of spots attributed to distinct conditions were performed with the paired t-test, followed by the FDR (False Discovery Rate) adjustment procedure. Asterisks denote significant differences according to the Wilcox test with p-values adjusted using the Benjamini-Hochberg procedure (* — p < 0.05, ** — p < 0.01, *** — p < 0.001).

We then summarized the mean intensities of the spots within the clusters. Since differences between the k-means clusters were less noticeable (**Supplementary Figures 10A, B**), we examined the hierarchical clustering of significant spots. This approach reflected the dynamics of protein production changes during anoxia and re-aeration (**Supplementary Figures 11A, B**). Certain spots showed higher intensity in control compared to anoxia and re-aeration, whereas others, conversely, increased during treatments (**Supplementary Figures 11A, B**). Importantly, changes within clusters corroborated the analysis of significant spots obtained using the Bayesian approach (**Supplementary Table 12**). Subsequent hierarchical clustering procedure on these selected spots results corroborated that anoxic and reoxygenation-associated proteomes form a single cluster (**Figures 4C, D**). Given that the hierarchical clustering procedure provides more congruent inferences, we might conclude that the proteomes of roots and shoots under anoxia and reoxygenation are considerably similar. Moreover, the clusters corroborated the presence of four protein groups relative to their abundance under distinct conditions as described in section 3.2 (**Figures 5A, B**; **Supplementary Table 13**). It is worth noting that two groups displaying the continuation of accumulation/decrease during reoxygenation, which was triggered by anoxia, constituted 10 and 6 proteins out of 13 and 8 in shoots and roots, thus reaching 76% of the annotations.

### 3.4 Functional annotation of the identified proteins

**Figure 6:**
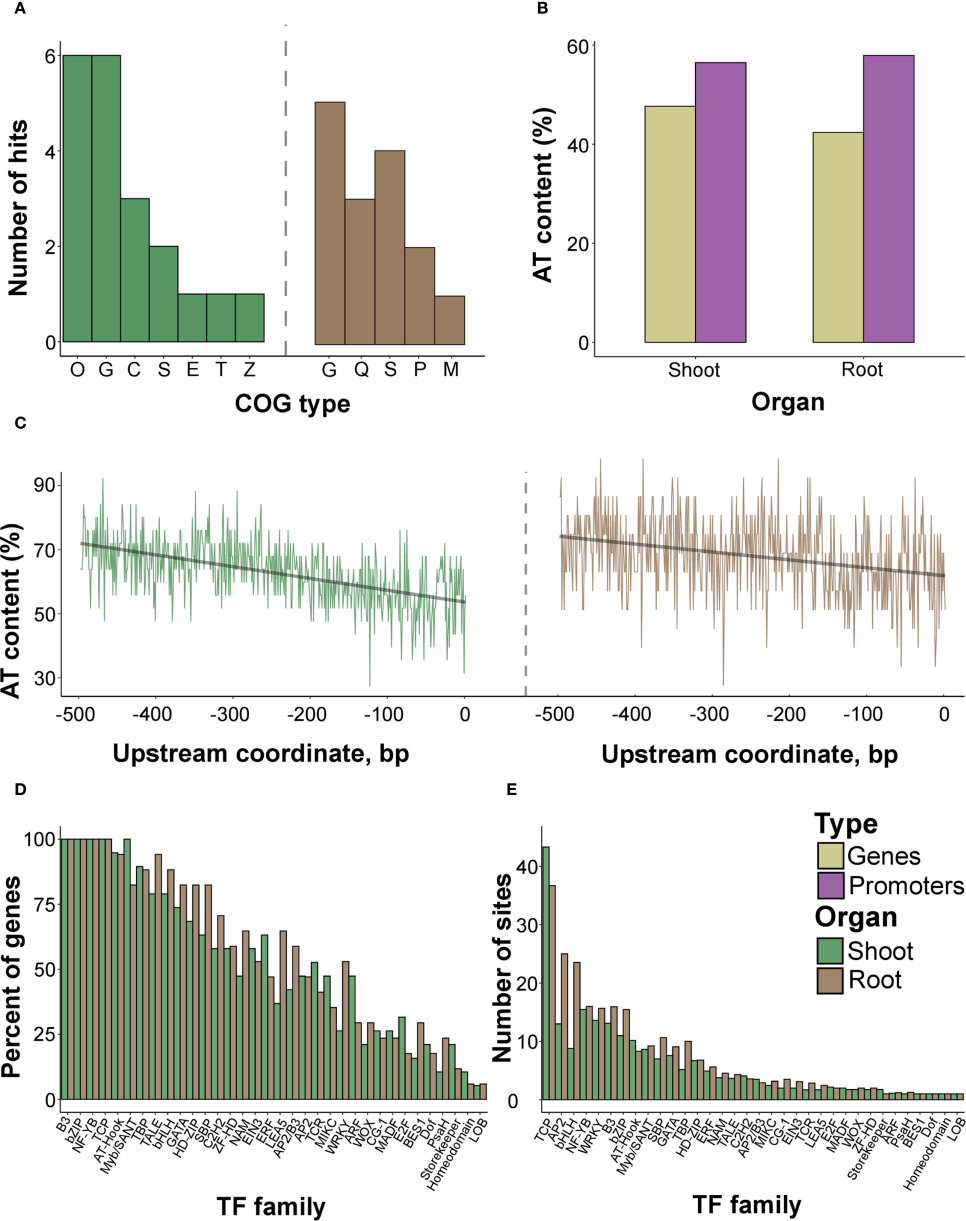
Functional analysis of the sequences of the identified proteins and genes encoding them. **(A)** The composition of COG (Cluster of Orthologous Genes) terms in protein sequences attributed to the root and shoot proteomes. **(B)** AT content in genes coding for the analyzed proteins from shoot and root proteomes, as well as their upstream 500 bp regions. The exact numbers are provided in **Supplementary Table 17**. **(C)** The per-site distribution of AT-content in the sequences of upstream 500 bp regions of loci encoding found proteins. **(D)** The percentage of genes possessing at least one predicted binding site of a particular TF family encoding annotated proteins from shoots and roots. **(E)** The mean number of predicted binding sites of a particular TF family in the respective loci.

To reveal the functional characteristics of the identified proteins, we characterized the distribution of COG (Cluster of Orthologous Genes) and GO (Gene Ontology) terms based on protein sequences. Proteins from both organs were enriched with the G (carbohydrate transport and metabolism annotation) category (**Figure 6A**). Shoot proteins were associated with O (posttranslational modification, protein turnover, chaperones) and C (energy production and conversion) categories. In contrast, for root proteins, the most abundant terms were Q (secondary metabolites biosynthesis, transport, and catabolism) and P (inorganic ion transport and metabolism). The GO overrepresentation test failed to identify significant annotations using both all and significant spots (**Supplementary Table 14**), with only one putative (p-value = 0.07) association (hydrolase activity, GO:0016787) assigned to the proteins of rice roots.

### 3.5 Analysis of *cis*-elements in promoter regions

To reveal possible regulatory mechanisms, we analyzed the upstream regions of genes encoding the identified proteins. Noteworthy, the genes were evenly distributed among the chromosomes (**Supplementary Figure 12A**), especially in the genes coding for metabolic proteins of shoots (**Supplementary Table 15**) and components of the antioxidant system in roots (**Supplementary Table 16**). These findings indirectly indicate common regulatory pathways governing gene expression during stress conditions.

Next, we excised 500 bp upstream sequences of the respective genes to analyze putative TFs with the closest proximity to transcription start regions as commonly reported for stress-responsive proteins (Doherty et al., 2009; Xu et al., 2015; Shani et al., 2017). The AT content in the upstream sites exceeded the protein-coding regions (**Figures 6B, C**, **Supplementary Table 17**). Having collected the predicted TFBSs using the PlantPAN utility (Chow et al., 2019), we revealed that the number of sites strongly correlated with the number of TF families attributed to the genes (p-value< 2.2e-16 according to the Pearson test) (**Supplementary Figures 12B–D**). Since the relationships were preserved when using all genes or those corresponding to significantly different proteins (p-value = 4.769e-06 according to the Pearson test, **Supplementary Figures 12E, F**), we further considered all genes. We observed three distinct maxima ot the TFBSs within the -400, -250, and -50 bp. upstream regions (**Supplementary Figures 13A–C**, **14A–C**).

**Figure 7:**
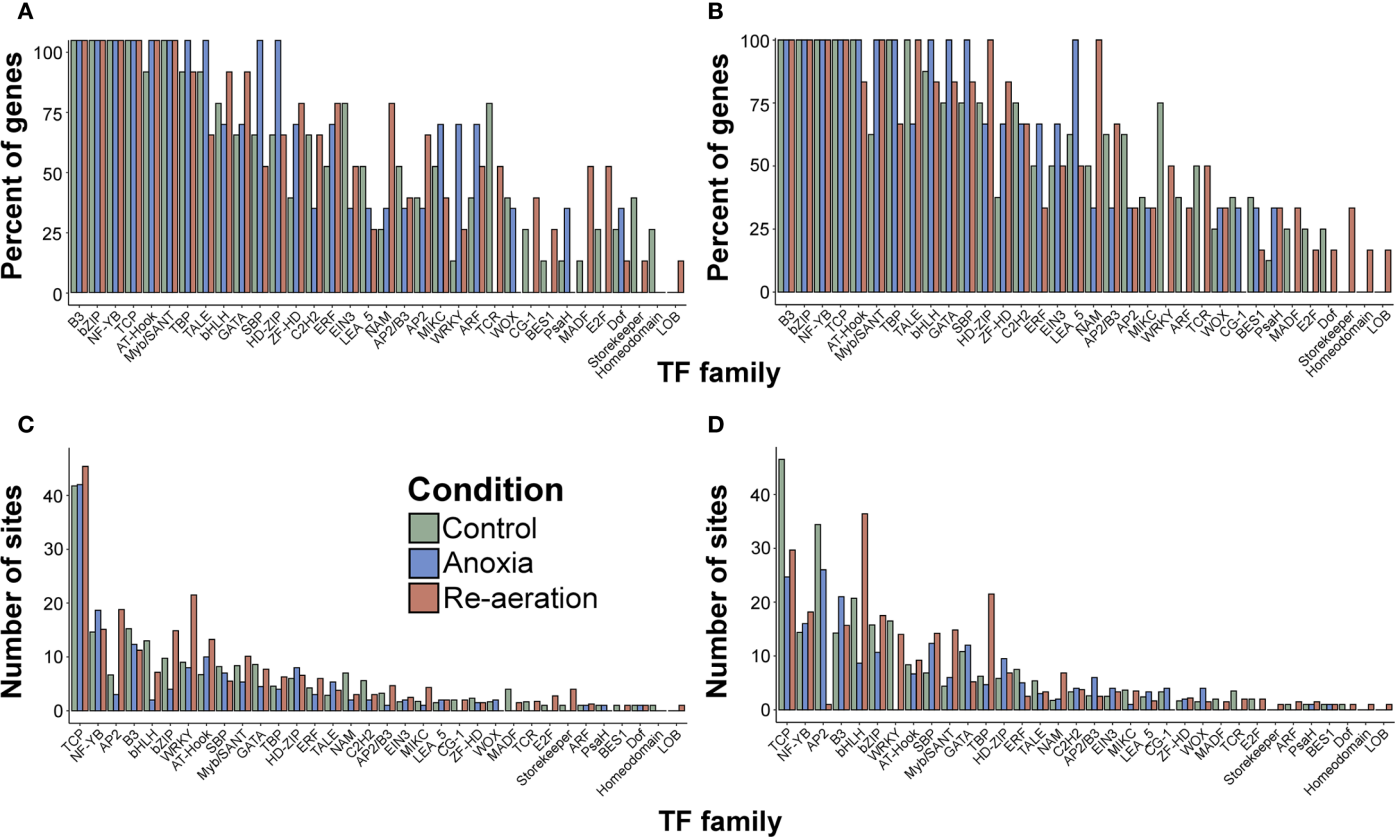
The percentage of genes encoding annotated proteins from shoots **(A)** and roots **(B)** with at least one TFBS of a certain TF family, grouped according to the condition in which the relative intensity calculated with the PDQuest software of the respective spots was the highest. The mean number of sites of a certain TF family in genes encoding proteins identified with mass spectrometry from shoots **(C)** and roots **(D)** classified as described above. See **Supplementary Table 18** for further details.

The differences between genes attributed to rice organs, considering the percentage of loci possessing TFBSs of distinct TF families, were generally negligible (**Supplementary Table 18**, **Figure 6D**. The mean number of TFBSs was similar as well, except for TCP, AP2, and bHLH, with more sites in root-associated genes (**Figure 6E**). We then grouped the examined genes condition-wise according to the highest intensity of the spots harboring protein products under certain treatments. The overall percentage of TFBSs within promoters resembled the inferences from organ-specific comparisons, with uniform distributions. The contrast was visible for genes encoding proteins accumulated during anoxia (WRKY, HD-ZIP, SBP) and re-aeration in shoots (NAM, MADF, TCR, E2F, ERF) (**Figure 7A**; **Supplementary Table 19**). In roots, the analogous stark contrast was observed in loci associated with response to anoxia (LEA_5, ERF) and re-aeration (NAM, ZF-HD, HD-ZIP) (**Figure 7B**; **Supplementary Table 19**).

The most evident variance was related to the average number of TFBSs for each condition-attributed gene group. In rice shoots, loci encoding proteins found in control settings belonging to WRKY, AP2, bZIP, AT-Hook, Myb/SANT, and ERF; re-aeration-associated genes contained more TFBSs of bHLH, NAM, and GATA, while genes associated with proteins accumulating during anoxia were enriched with NF-YB (**Figure 7C**; **Supplementary Table 19**). In roots, the following distribution was obtained (for simplicity, only the condition-based group is listed): (i) (TCP, WRKY, AP2, and ERF), (ii) re-aeration (bHLH, TBP, Myb/SANT, SBP, and bZIP), and (iii) anoxia (GATA) (**Figure 7D**; **Supplementary Table 19**). In all the genes analyzed, the TCP family exhibited the highest number of sites on average.

Next, we compared the homologous loci using the same condition-based grouping in *A*. *thaliana*. It is known for moderate tolerance and a distinct response to anoxic conditions in comparison to rice (Narsai and Whelan, 2013). Notably, differences between genes attributed to the distinct organs were less noticeable (**Supplementary Figure 15A**; **Supplementary Table 20**). The only visible difference referred to the abundance of sites related to small RNAs in loci associated with shoots (**Supplementary Figure 15B**). The dissimilarities in the percentage of genes annotated with TBBSs included those related to anoxia re-aeration (TCP and HD-KNOTTED) in shoots (**Supplementary Figure 16A**) as well as those associated with anoxia (WRKY(Zn), ABI3/VP1, HD-PHD) and re-aeration (HD-KNOTTED, bHLH, small RNAs, and TCP) in roots (**Supplementary Figure 16B**). In terms of the number of sites, only re-aeration-associated genes in shoots displayed TFBSs within small RNAs and the AT-Hook TF family (**Supplementary Figures 16C, D**).

## 4 Discussion

### 4.1 Rice shoots synthesize proteins with antioxidant properties during anoxia and re-aeration to neutralize reactive oxygen species in stress conditions

Using the 2-DIGE, we characterized the proteomes of rice shoots and roots under anoxia and re-aeration and distinguished four protein groups according to condition-wise abundance (**Figure 5A**). The first group encompassed those slightly or drastically decreasing during anoxic exposure and subsequent reoxygenation. These were enzymes of primary metabolism and photosynthetic components. Enhanced synthesis of fructose-bisphosphate aldolase (FBPase) was reported in rice coleoptiles (Huang et al., 2005) and *Acorus calamus* (Bucher and Kuhlemeier, 1993) in short-term anoxia, with a subsequent decline during prolonged anoxia. The FBPase decrease is accompanied by the synthesis of nucleoside diphosphate kinase (NDPK), maintaining cell homeostasis and integrity via obtaining ATP from the sucrose in tandem with sucrose synthase (Guglielminetti et al., 1995; Stitt, 1998). We found only a slight reduction of the FBPase in anoxia, followed by a visible decline during reoxygenation, indicating a switch to respiration when oxygen is available. We have not observed NDPK in our proteome, since it was detected after 2–3 d of anoxic treatment (Guglielminetti et al., 1995; Cui et al., 2005); thus, we possibly examined a transitory state when FBPase starts to decrease and NDPK is not synthesized considerably yet. We propose two possible reasons to explain the behavior of the small subunit of RuBisCO (SSU). First, it might decrease due to its proteolysis to provide amino acids for protein turnover in dark conditions. Second, the high rate of ROS generation could impair RuBisCO assembly. Such a response was reported in rice (Chen et al., 2019), *Solanum lycopersicum* (Ahsan et al., 2007), and *Medicago sativa* (Zeng et al., 2019) under oxygen depletion. The reduction of SBPase (sedoheptulose-1,7-bisphosphatase) and PRK (phosphoribulokinase) in re-aeration may be explained by the elevated ROS levels. The content of SBPase declined in *Physcomitrella patens* (Kukuczka et al., 2014) and *Glycine max* (Nanjo et al., 2013) subjected to anoxia and post-flooding, respectively. On the contrary, the protein accumulated in *A*. *thaliana* (Yang et al., 2022) and a tolerant mutant of *G. max* (Yin et al., 2016) after long-term anoxia and flooding. Similarly, waterlogging caused a reduction in PRK level in *Zea mays* (Yu et al., 2015) and *Sesamum indicum* (Jung et al., 2019).

The second group of spots reached a peak during anoxia, followed by a dramatic drop after 24 h of re-aeration. Unlike SSU, the large subunit of RuBisCO (LSU) accumulated in anoxia and was considerably reduced only after 24 h of re-aeration. A similar pattern was detected in bean leaves (Hildbrand et al., 1994), *Pyrus communis* (Pedreschi et al., 2009), and *Rhizophora mucronata* (Piro et al., 2023). However, more frequently its level is reduced as shown for *S. indicum* (Jung et al., 2019), *S. lycopersicum* (Ahsan et al., 2007), and *Triticum aestivum* when exposed to waterlogging (Pan et al., 2019). Therefore, these studies and our results support that LSU may serve as a hub of amino acids that can be efficiently reutilized when needed. Our observations indicate that the demand for protein synthesis during re-aeration may be satisfied by LSU degradation. Heat shock proteins of the HSP70 family are well-known for their ubiquitous role in mitigating various unfavorable conditions (Ul Haq et al., 2019). Importantly, HSP70 proteins are involved in resistance against anoxic stress in Arabidopsis (Banti et al., 2008), as well as in maize and soybean plants (Chen et al., 2014; Ul Haq et al., 2019). Distinct *Hsp70* transcripts showed dissimilar up- and down-regulation during anoxia and other stresses in rice (Sarkar et al., 2013) and *G. max* (Kong et al., 2010; Otori et al., 2021).

The third group of proteins includes those that started to increase during anoxia and reached a peak in reoxygenation. The photosynthetic NDH subunit of lumenal location 5 is a part of the NADH dehydrogenase complex located on the chloroplastic membranes (Vinogradov and Grivennikova, 2016). It is considered to maintain photosystem I through inducing CEF (Cyclic Electron Flow), which is crucial during stress (Kukuczka et al., 2014). In oxygen depletion, NDH components were synthesized in *P. patens* (Kukuczka et al., 2014) and *M. sativa* (Zeng et al., 2019). Transketolase (TK) takes part in the Calvin cycle as well as in the pentose phosphate pathway (PPP), converting fructose 6-phosphate into pentose-5 phosphate, linking glycolysis with PPP (Lakshmanan et al., 2014). The sufficient increase of TK content was illustrated by computational predictions of rice transcriptomics data (Lakshmanan et al., 2014) and experimental data in *Pyrus communis* (Pedreschi et al., 2009), *A*. *thaliana* (Yang et al., 2022), and *T. aestivum* (Pan et al., 2019) under anoxia and waterlogging. The abovementioned observations imply the key metabolic role of TK during anoxia and reoxygenation (Chirkova, 1998; Chirkova and Yemelyanov, 2018). Oxygen-evolving enhancer protein (OEE), consisting of three subunits, is bound to photosystem II (PSII), mediating the photolysis of water (Ahmad et al., 2017). The protein is positively associated with the response to oxygen reduction in plants, including rice (Wang et al., 2021), barley (Luan et al., 2018), and maize (Chen et al., 2014). OEE2 is easily detached from PSII, hence the stability of the complex may be restored by increased abundance of the protein (Ahmad et al., 2017). Importantly, OEE1/2 production is associated with the activity of antioxidant enzymes (Ali et al., 2018). OEE2 was hypothesized to be a part of the plant signaling system regulating the redox balance of the cell (Yang et al., 2003). Since oxidized OEE2 could be removed from PSII, the dual role of this protein may be proposed, i.e., the stabilization of PSII, and the involvement in the regulatory stress-mediated system, which is corroborated by our findings. This hypothesis is consistent with enrichment with lysine residues in OEE1/2 (**Figure 3F**). Lysins are susceptible to carbonylation, which could activate OEE1/2-triggered redox signaling. The stromal HSP70, unlike the aforementioned counterpart, sufficiently increased during re-aeration, corroborating the dissimilar expression profiles of *Hsp70* genes (Sarkar et al., 2013). Chaperonin 20 assists chaperonin 60 kDa in the folding of chloroplast proteins (Nazari et al., 2020). Noteworthy, it is also responsible for activating antioxidant enzymes (Kuo et al., 2013) and was up-regulated in rice (Wang et al., 2021) and a resistant mutant of soybean (Yin et al., 2016) under anoxia, while in other cases the latter showed significant down-regulation (Komatsu et al., 2013).

The final group encompassed proteins with a distinct behavior decreasing in anoxia and sufficiently accumulating during reoxygenation. The 2-Cys peroxiredoxin BAS1 protein is associated with the photosynthetic apparatus, protecting it from oxidative damage (Baier and Dietz, 1999) through binding to unsaturated fatty acids, thereby preventing lipid peroxidation (Baier and Dietz, 1997; Broin and Rey, 2003). Although its abundance has not been studied under the re-aeration exposure, it was shown to be down-regulated in *G. max* (Otori et al., 2021), *Acanthus mollis* (Liu and Zheng, 2021), and *Cicer arietinum* (Komatsu et al., 2021) when exposed to flooding of varying durations, thus probably implying its primary role during re-aeration.

### 4.2 The proteome of rice roots under anoxia and re-aeration is enriched with components of plant immunity

In roots, the first group included fructose-bisphosphate aldolase 3 and peroxidase P7. We proposed that the changes in FBPase content are explained by the same mechanisms that occur in shoots, indicating a metabolic switch to respiration. In banana plants, peroxidase P7 is localized in the plasma membrane and tonoplast and protects tolerant cultivars from lipid peroxidation under cold stress (Gao et al., 2021). However, a recent study shows that in rice roots, during anoxia, peroxidase in the cytoplasm decreases, while elevated activity levels were detected for both apoplastic and cytoplasmic isoforms upon re-aeration (Yemelyanov et al., 2022), indicating unequal responses of plant organs to stress conditions. The behavior of antioxidant systems in plants under anoxia is not uniform. In tolerant species, a slight decrease or negligible accumulation of antioxidants during anoxia and re-aeration was reported (Ushimaru et al., 1992; Biemelt et al., 1996). Our recent results showed the restoration of the activity of enzymes of the ascorbate-glutathione cycle and the up-regulation of corresponding genes in rice upon re-aeration (Yemelyanov et al., 2024). These studies and current findings illustrate that stress-tolerant species could already possess sustainable redox status-managing systems, whereas sensitive species accumulate these components to withstand oxidative stress.

Prb1 (a putative pathogenesis-related protein) contains a conserved SCP domain common for pathogenesis-related proteins (PR) (Marchler-Bauer et al., 2007) and has a similar function to PR1 (Sessa et al., 1995). Due to its function, Prb1 is activated during biotic stress, but is was detected in wheat after waterlogging (Pan et al., 2019) and rice during drought-flood alternation stress (Xiong et al., 2019). We hence speculate that the Prb1 protein may mediate the initial phase of plant reaction to stresses. The decrease during re-aeration could indicate a specific response to oxygen depletion only.

PR-1a protein fell into the third group, which steadily increased during anoxia and re-aeration. Apart from biotic stress, it is accumulated in low-oxygen environments in rice (Xiong et al., 2019), wheat (Pan et al., 2019), soybean (Lin et al., 2019), and *P. patens* (Kukuczka et al., 2014). Notably, PR-1 was accumulated under re-aeration in *S. lycopersicum* (Czernicka et al., 2022). Another component of plant immunity, chitinase 2, is also observed in the proteomic landscapes of plants subjected to submergence and waterlogging, such as *Brassica napus* (Xu et al., 2018), *O. sativa* (Xiong et al., 2019), *Hordeum vulgare* (Andrzejczak et al., 2020), *T. aestivum* (Pan et al., 2019), and *Z. mays* (Yu et al., 2015). Cysteine-rich receptor-like kinases (CRKs) regulate symbiotic and parasitic relationships (Quezada et al., 2019), while they were found in cucumber (Xu et al., 2016) and rice (Xiong et al., 2019) when subjected to waterlogging and drought–flood alternation, respectively. Similar to other discussed proteins, glucan *endo*-1,3-β-glucosidase is involved in response to pathogen-caused lesions (Beffa and Meins, 1996) and also associated with low oxygen treatment of rice (Wang et al., 2021), maize (Chang et al., 2000), and wheat (Komatsu et al., 2022). It should be noted that both biotic interactions and unfavorable conditions launch a cascade of redox reactions in which ROS take part (Mittler, 2017). Taking into account the aforementioned data, we might conclude that PR-1a, chitinase 2, CRK 6, and glucan *endo*-1,3-β-glucosidase in rice represent universal stress proteins probably involved in stress-induced signal transduction and adaptation to pathogen attacks that could accompany reoxygenation during post-flooding in nature.

The enzyme α-*L*-arabinofuranosidase 1 (ABF1) belonged to the fourth group. It catalyzes the degradation of carbohydrates tied to arabinogalactan-proteins (AGPs), controlling the reconstruction of the cell wall (Chávez Montes et al., 2008; Kotake et al., 2006). Similar to BAS1 peroxiredoxins in shoots, ABFs tend to decrease in rice (Xiong et al., 2019) and soybean under flooding (Lin et al., 2019). It, therefore, can be hypothesized that ABF1 plays a dual role in both releasing carbohydrates to meet energy demand and maintaining cell wall integrity.

### 4.3 The adaptive mechanisms of plants during re-aeration remain largely unstudied, but the existing reports and current findings imply a universal response to reoxygenation

Despite agricultural significance, there is still an evident skew towards studying oxygen depletion, but not re-aeration. Having collected the information based on 99 proteomic articles, we found that only 7 included the re-aeration period (**Supplementary Table 21**). Proteomics inferences showed that rice subjected to hypoxia/anoxia accumulates glycolytic enzymes, LEA, peroxiredoxins, SOD, HSPs, PR proteins, and signaling cascade components and demonstrates a considerable reduction in certain enzymes, such as ascorbate peroxidase, malate dehydrogenase, glutathione S-transferase, and glycosyltransferase (Huang et al., 2005; Sadiq et al., 2011; Shingaki-Wells et al., 2011; Chen et al., 2019). A combination of submergence and low temperature or drought showed a similar proteomic landscape as described here, with the induction of OEE2, HSP70, glucan *endo*-1,3-β-glucosidase, extension, ADH1, proteins related to plant immunity (PR proteins, CRK 6, chitinase, etc.), and concomitant down-regulation of enzymes, namely, phenylalanine ammonia-lyase, glutathione S-transferase, and malic enzyme (Xiong et al., 2019; Wang et al., 2021).

The present body of studies related to the re-aeration period suffices to provide insights into how plants adapt to reoxygenation. When oxygen returns, the plant switches from anaerobic fermentation to respiration. In rice mitochondria, cytochromes and Fe-S clusters were synthesized during re-aeration, while the content of aldolase, conversely, decreased (Millar et al., 2004). A continued re-aeration period can be perceived as priming, as reported for peptidyl-prolyl *cis*-*trans* isomerase and osmotin in *S. lycopersicum* accumulating during post-waterlogging and the repeated waterlogging as well (Czernicka et al., 2022). Other research items were carried out on a model object, the soybean, subjected to flooding with subsequent recovery. The post-flooding proteome included subtilisin-like serine endopeptidase, HAD acid phosphatase, and antioxidant enzymes, coupled with reduced content of ribosomal proteins and eIF2beta subunit (Khan et al., 2014). 2Fe-2S ferredoxin family protein, nucleotidylyl transferase, pyruvate kinase, and β-ketoacyl reductase were up-regulated at the 4–6 d recovery stage, followed by reduction after 8 d of post-flooding (Khan et al., 2015). The severity of the stress determines the proteomic picture. Flooding treatment for 3 d followed by 2 d recovery increased the content of Alcohol dehydrogenase-1F and down-regulated annexin, whereas 1 d flooding with 4 d of re-aeration gained the opposite results. Moreover, in the former case, more signals related to plant immunity, such as PR 10, were observed (Salavati et al., 2012). In the joint analysis of roots and hypocotyls, irrespective of the treatment duration, elevated levels of polygalacturonase-inhibiting protein 1 and expansin with down-regulated ribosomal proteins and S-adenosyl-*L*-methionine-dependent methyltransferase were observed (Yasmeen et al., 2016). At the same time, the content of the former in hypocotyls only decreased after a severe flooding and recovery period (Nanjo et al., 2013).

The obtained proteomic landscape in rice shoots and roots treated with 24 h of anoxia and re-aeration gives evidence that these two exposures cause similar defense mechanisms according to clustering patterns of condition-specific samples (**Figures 4A, B, C, D**; **Supplementary Figures 3, 4, 5, 6**). Therefore, we conclude that re-aeration is more a ramification of anoxia with similar effects, corroborating that flooding and post-flooding response of soybean plants affected the same set of proteins differing only in quantity (Yasmeen et al., 2016). Here, only three proteins can be considered anoxia-specific, such as LSU, HSP70 2, and Prb1, whereas two represent reoxygenation-specific proteins, namely, BAS1 and ABF1, while 76% of annotations corresponded to proteins with the same continuing behavior in anoxia and reoxygenation. Therefore, it could be proposed that oxidative stress starts in anoxia and continues in re-aeration, further altering protein abundance. The enzymes of primary metabolism (cytoplasmic and plastid FBPase, SBPase, and PRK) are decreasing, indicating metabolic alterations. Conversely, systems involved in the defense of photosynthetic apparatus (NDH subunit 2, OEE1/2), protein stability (HSP70, chaperonin 20 kDa), plant immunity (PR-1a, CRK, chitinase 2), and cell-wall restructuring (*endo*-1,3-β-glucosidase) are activated.

Known studies generally show that anoxia and re-aeration induced the accumulation of proteins associated with glycolysis, cell wall reconstruction, immunity, and the decrease of proteins involved in photosynthesis and translation, whereas heat shock proteins and antioxidants had the dual behavior (**Supplementary Table 22**). The overall similarity between the existing reports and our data reached 57-74%, depending on the plant and organ (**Supplementary Figure 17A–D**; **Supplementary Tables 23, 24**). For individual proteins, the most similar group was those that started accumulating during anoxia and reached a peak in the reoxygenation period (**Supplementary Table 25**). This observation further proves that a universal adaptation to recovery from stress involves general strategies. Therefore, we assume that during anoxia rice becomes pre-adapted to further re-aeration-induced oxidative stress and putatively to pathogens, ensuring successful survival during the post-anoxic period in nature.

### 4.4 Transcriptional regulation of stress-responsive genes during anoxia and re-aeration is likely not confined to the ERF-VII group and unites multiple TF families

We analyzed the number of TF binding sites (TFBSs) in genes encoding condition-specific proteins, since it is proportional to promoter strength (Puig et al., 2021) and might reflect the regulatory network. To date, tremendous progress has been made in our understanding of how plants respond to oxygen depletion in the context of transcriptional regulation. Transcription factors of the ERF family, and in particular the ERF-VII group, are the most well-studied players triggering two different strategies of plant tolerance to submergence, low-oxygen escape syndrome (LOES) and low-oxygen quiescence syndrome (LOQS) (Bailey-Serres and Voesenek, 2008; Nishiuchi et al., 2012; Voesenek and Bailey-Serres, 2013) via activating SK (SNORKEL1/2) or SUB1 (SUBMERGENCE 1A/B/C), respectively (Loreti and Perata, 2023). Other known factors included representatives of ERFs, i.e., HRE1, HRE2, RAP2.2, RAP2.12, and RAP2.3 (Voesenek and Bailey-Serres, 2013), LBD41 (Jethva et al., 2022), and WRKY33/WRKY12 (Tang et al., 2021), HSFA2 (Banti et al., 2010), ANAC017 (Bui et al., 2020), and almost all plant TF families, such as zinc fingers, MYB, NAC, bHLH, bZIP, homeobox, etc (Licausi et al., 2011).

We revealed that NF-YB and GATA were associated with anoxia (**Figure 7C, D**). Nuclear factor Y (NF-Y) is a conserved TF complex of three subunits (A, B, and C) that mostly but not exclusively binds genes encoding proteins engaged in photosynthesis (Stephenson et al., 2010). Both up- and downregulation of NF-Y components were reported during waterlogging (Teoh et al., 2022), submergence (Leyva-González et al., 2012; Müller et al., 2021), and pure anoxia (Lasanthi-Kudahettige et al., 2007). Similarly, GATA factors are conserved proteins regulating plant development and response to environmental stimuli (Zhang et al., 2023b). The expression of multiple genes from this family was impacted by low oxygen exposure in *A. thaliana* tolerance (Van Veen et al., 2016), *B. napus* (Liu and Zwiazek, 2022), *Cucumis sativus* (Kęska et al., 2021), and other plants.

Interaction networks of TFs in re-aeration are less understood. According to current views, ERF-VII factors can be perceived as integrated regulators modulating gene expression during returning to normoxia as well. When oxygen is unavailable, the N-terminal pathway of degradation is repressed, leading to the accumulation of ERF-VIIs, which triggers anaerobic transcription (Voesenek and Bailey-Serres, 2015), while subsequent re-aeration induces degradation of ERF-VII factors, thus enabling a switch to normoxia (Voesenek and Bailey-Serres, 2015; Giuntoli and Perata, 2018). As the recovery during re-aeration occurs under a combination of stress conditions, multiple TFs serve to combat such adverse effects. These included HaWRKY76 (Raineri et al., 2015) associated with dehydration, WRKY22 contributing to plant immunity (Hsu et al., 2013), MYC2 related to oxidative stress (Yuan et al., 2017), and a whole array of transcription factors, such as NAC, MYB, bZIP, zinc fingers, LOB, HD-ZIP, HSF, and others (Li et al., 2022).

We found that genes coding for re-aeration-specific proteins were enriched with TBP and SBP binding sites (**Figure 7C, D**). TATA-box binding proteins (TBP) with TBP-associated factors constitute TFIID, a general transcription factor in eukaryotic organisms (Zhang et al., 2020).TBP factors were affected by re-aeration and de-submergence in *A. thaliana* (Yao et al., 2017) and *G. max* (Tamang et al., 2014). The SBSs (*SQUAMOSA* promoter binding protein) are highly conserved proteins regulating both development and adaptive response to stressors (Wang et al., 2018), including reoxygenation, as was demonstrated for *A. thaliana* (Yuan et al., 2017), *O. sativa* (Locke et al., 2018), *G. max* (Tamang et al., 2021), and *C*. *sativus* (Kęska et al., 2021). While we could not attribute TCPs to a certain condition, all studied genes were enriched with TCP-related signals, highlighting their versatile functions in stress and normal plant life. Accumulated evidence shows that TCPs are affected by re-aeration in *Paeonia ostii* (Zhang et al., 2022), *Rhododendron delavayi* (Zhang et al., 2023a), *A. thaliana* (Yuan et al., 2017), *O. sativa* (Locke et al., 2018), *G. max* (Tamang et al., 2021), and *C*. *sativus* (Kęska et al., 2021). Such observations, coupled with our data, suggest that TCP members are prominent targets to improve crop tolerance.

In contrast to rice, we revealed noticeable signals within homologous sequences from Arabidopsis only in terms of undetermined small RNAs in genes attributed to re-aeration (**Supplementary Figure 16B**). It was shown that different small RNAs, namely, TAS1 and TAS3 families, promote plant tolerance to hypoxia during waterlogging and submergence (Moldovan et al., 2010; Betti et al., 2020).

Given that the proteomes under anoxia and re-aeration were similar, we proceeded with scrutinizing the global landscape of TF families responding to oxygen depletion and return to normal conditions to reveal common regulatory pathways based on 326 research articles (**Supplementary Table 26**). We found a significant overlap between distinct stressors and plants (**Supplementary Figures 18A, B, C**), reflecting the universal nature of conditions impacting TFs. Importantly, the co-occurrence graphs of TFs activation under anoxia and re-aeration were identical (**Supplementary Figures 19A, B**). These patterns reaffirm that transcriptional regulation programs orchestrating plant adaptation to anoxia and re-aeration are quite alike. On that account, we might assume that the accumulation of gene transcripts that are crucial for plant survival during the re-aeration starts in anoxic conditions.

Moreover, we found that certain proteins, such as OEE2, are enriched with lysine. Lysine residues are major targets of protein carbonylation (Davies, 2005). While this modification is considered damaging, in some cases, carbonylation can regulate diverse cellular processes (Johansson et al., 2004; Correa-Aragunde et al., 2015; Shikov et al., 2021a). Post-translational modifications represent a crucial regulatory mechanism that is activated under stress conditions and mediates plant responses (Vu et al., 2018; Kosová et al., 2021). Given that proteins with high lysine content represented either reoxygenation-associated or control proteins in shoots and roots, respectively, it can be proposed that, in addition to transcriptional regulation, oxidative post-translational protein modification may also take part in plant response to anoxia and re-aeration.

## 5 Conclusion

**Figure 8:**
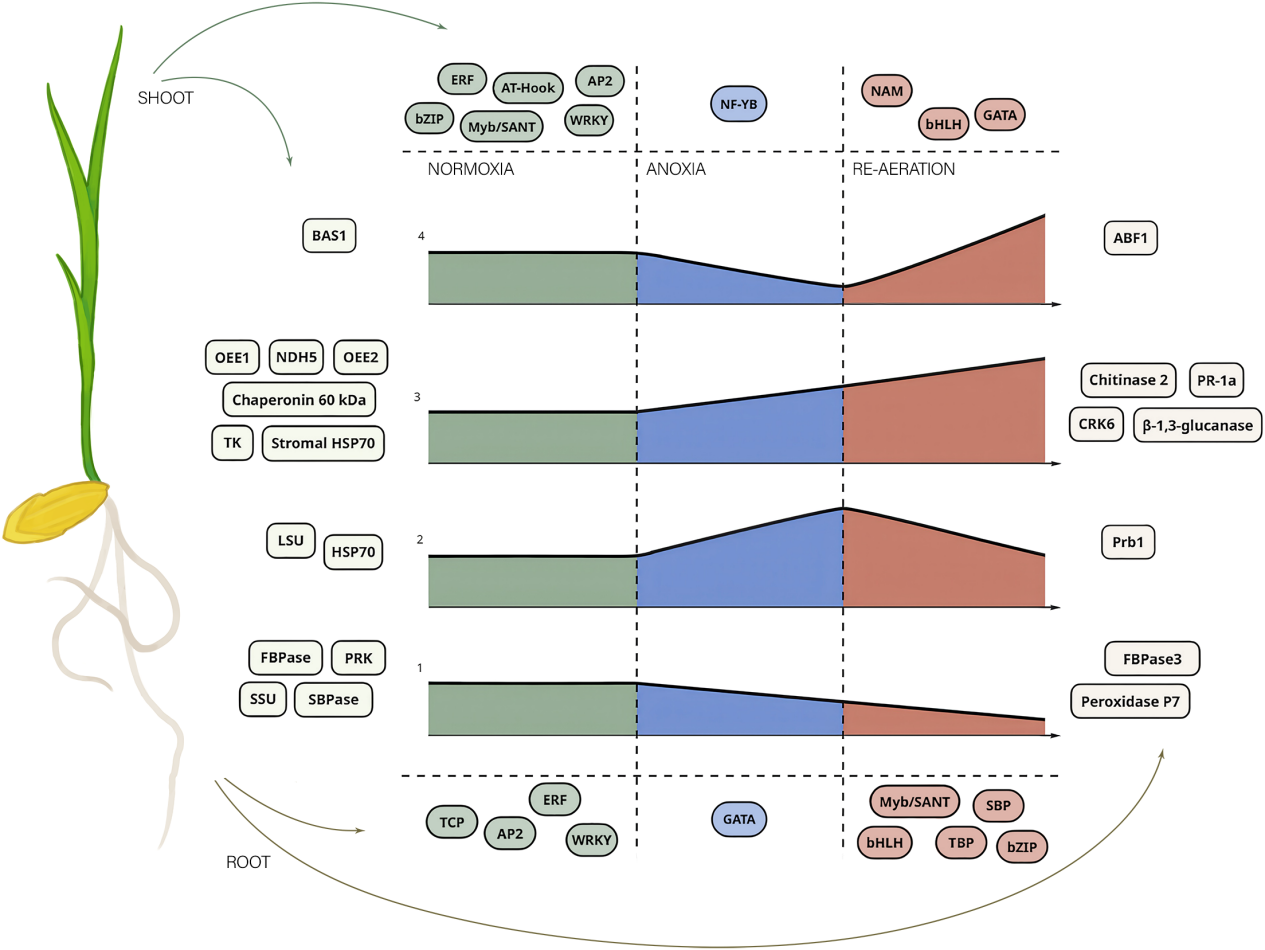
A general scheme of rice response to anoxia and re-aeration based on proteomics inference and bioinformatic predictions of TFBSs. The figure illustrates the key proteins attributed to certain conditions and putative TFs regulating gene expression of these moieties. The central graphs represent four types of proteins depending on their abundance (see **Figures 5A, B**). The X-axis of four subplots corresponds to the time of the exposure, while the size of the figure depicts the abundance of the protein product. The numbers from 1 to 4 indicate groups of proteins defined above according to the intensities of the spots containing them under distinct conditions. The numbers correspond to those presented in **Figures 5A, B**. The color of the areas encodes the treatment type. Shoot and root proteins are placed in rectangles on the left and the right sides, respectively, and colored according to the organ as well. The TFs associated with proteins related to the treatments are placed up- and downward to the plots for shoots and roots, respectively. Individual TFs are placed in the elliptic figures and are colored according to the condition.

Post-anoxia is a complex stress factor that is interconnected with oxygen depletion, which precedes the return to normoxic conditions. This stressor includes energy depletion and intense oxidative stress, desiccation, and susceptibility to pathogenic attacks. Therefore, to ensure survival in such deleterious conditions, rice plants accumulate a host of defensive proteins. The landscape of control, anoxic, and re-aeration proteomes showed that changes in protein abundance during oxygen depletion and re-aeration are sufficiently similar. Despite the presence of clear condition-attributed proteins, two main protein groups accounting for 76% of the identified moieties represent those that started to decrease during anoxia and continued to deplete after 24 h of re-aeration, and proteins that accumulated during stress conditions. It can be concluded that rice plants become pre-adapted to reoxygenation through the synthesis of proteins related to the antioxidant system, plant immunity, maintenance of photosynthetic apparatus, and protein folding (**Figure 8**). According to the distribution of transcription factor binding sites, this adaptation could involve a sophisticated network of transcriptional regulators. Importantly, all of the identified TFs belonged to stress-related families, especially those participating in plant response to drought, oxidative stress, and pathogenic attacks. We found several not thoroughly studied TFs, including those of families TBP, SBP, and TCP, involved in post-anoxic responses, possibly mediating the signaling in plants subjected to anoxia and re-aeration (**Figure 8**). Moreover, the enrichment of post-anoxic proteins with lysine residues susceptible to oxidation during re-aeration might indicate that oxidative post-translational regulation exists along with TF networks. While in this study, we modelled only the pure anoxic and reoxygenation exposures, proteomic approaches look promising to increase our knowledge of how plants can tolerate the post-anoxic period, representing both a direct continuation of anoxia and increased susceptibility to other stressors. However, only a few studies focused on reoxygenation have been done; thus, we are still far from a deep understanding of molecular changes in plants during re-aeration. Apparently, the progress in our understanding of how plants combat a combination of stressors during post-anoxia in nature is incremental. The mechanisms that are activated during oxygen depletion, as well as the causes of the deleterious effects of this condition, have been analyzed for more than a century. To date, we have become closer to unraveling reoxygenation-specific phenomena. Here, we applied the gel-based 2-DIGE technique, which allowed us to reveal only the key players controlling the adaptive strategy of rice plants while providing important insights. To analyze the impact of re-aeration per se, we excluded all possible factors, including light, which exists under natural conditions. However, we believe that analyzing complex factors would certainly require preliminary investigations of distinct stressors. Due to the practical and fundamental significance of this area of research, multi-omics studies with higher resolution are required to unveil the intricate networks of regulatory pathways. It is especially crucial to conduct comparative studies including species with different tolerance to anoxia and subsequent reoxygenation.

## Data Availability

All results obtained in this study, including raw sources, are available in the article and Supplementary Material. All scripts used in this work for data analysis and generating figures, coupled with the processed results of MS/MS analysis, are available at the GitHub repository: https://github.com/anton-shikov/Rice_proteomics. The mass spectrometry proteomics data have been deposited to the ProteomeXchange Consortium via the PRIDE (Perez-Riverol et al., 2025) partner repository with the dataset identifier PXD067255 and 10.6019/PXD067255.
