# Supplementary material for: Forewarned is forearmed: rice plants develop tolerance to post-anoxia during anoxic conditions by proteomic changes
Source: Front Plant Sci. 2025 Sep 30;16:1647411. doi: 10.3389/fpls.2025.1647411 (PMC12518287; doi:10.3389/fpls.2025.1647411)
Supplement: Supplementary file 1 [file DataSheet1.pdf]

Supplementary Material for:

Forewarned is forearmed: Rice plants develop tolerance to post-anoxia during anoxic conditions by proteomic changes

Shikov A.E. [1,2], Shost V.I. [1], Chirkova T.V. [1], Shishova M.F. [1], Yemelyanov V.V. [1,*]

[1]Faculty of Biology, St. Petersburg State University (SPbSU), 199034, Universitetskaya em., 7/9, St. Petersburg, Russia;

[2]Laboratory for Proteomics of Supra-Organismal Systems, All-Russia Research Institute for Agricultural Microbiology (ARRIAM), 196608, Podbelsky chausse 3, Pushkin 8, St. Petersburg, Russia.

*** Correspondence:**
Yemelyanov V.V.
bootika@mail.ru

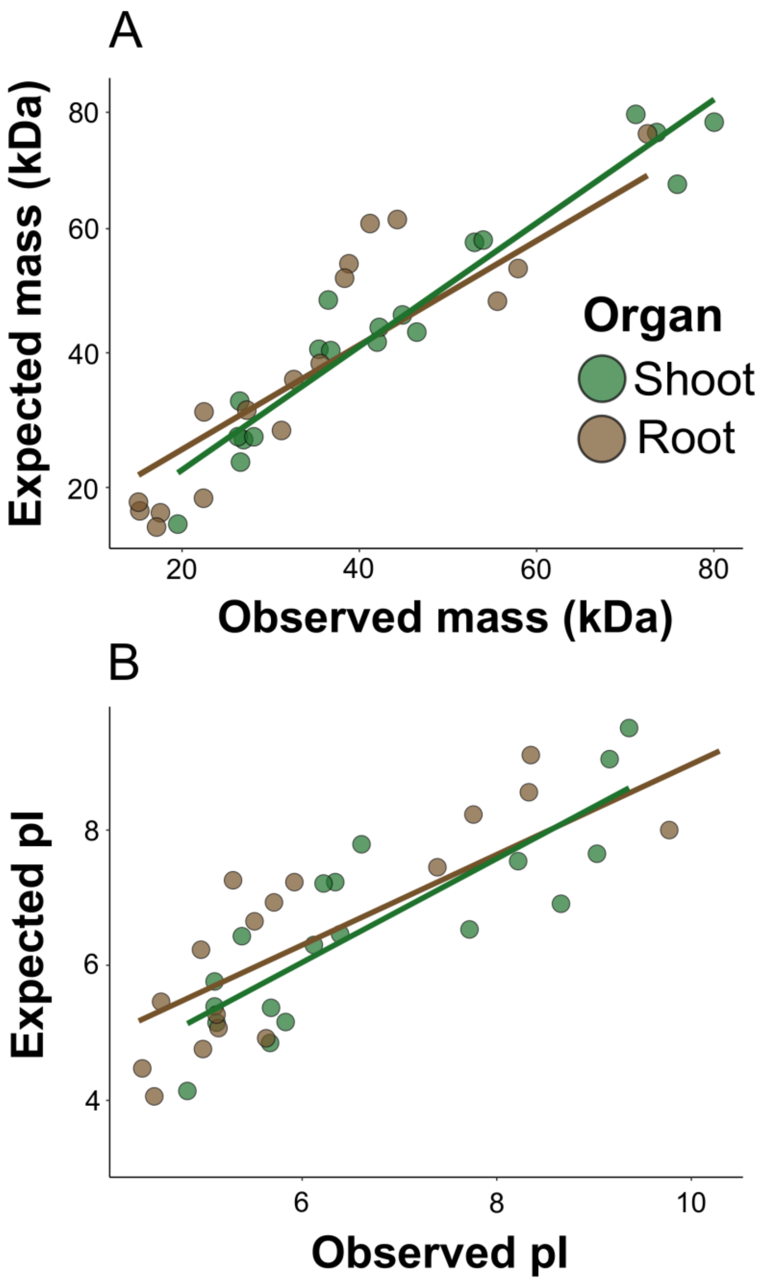

Supplementary Figure 1. (**A**) The correspondence between predicted and experimentally derived protein mass of identified proteins. (**B**) The same data for the isoelectric points (pI) of the respective proteins. The exact numbers are provided in **Supplementary Table 4**.

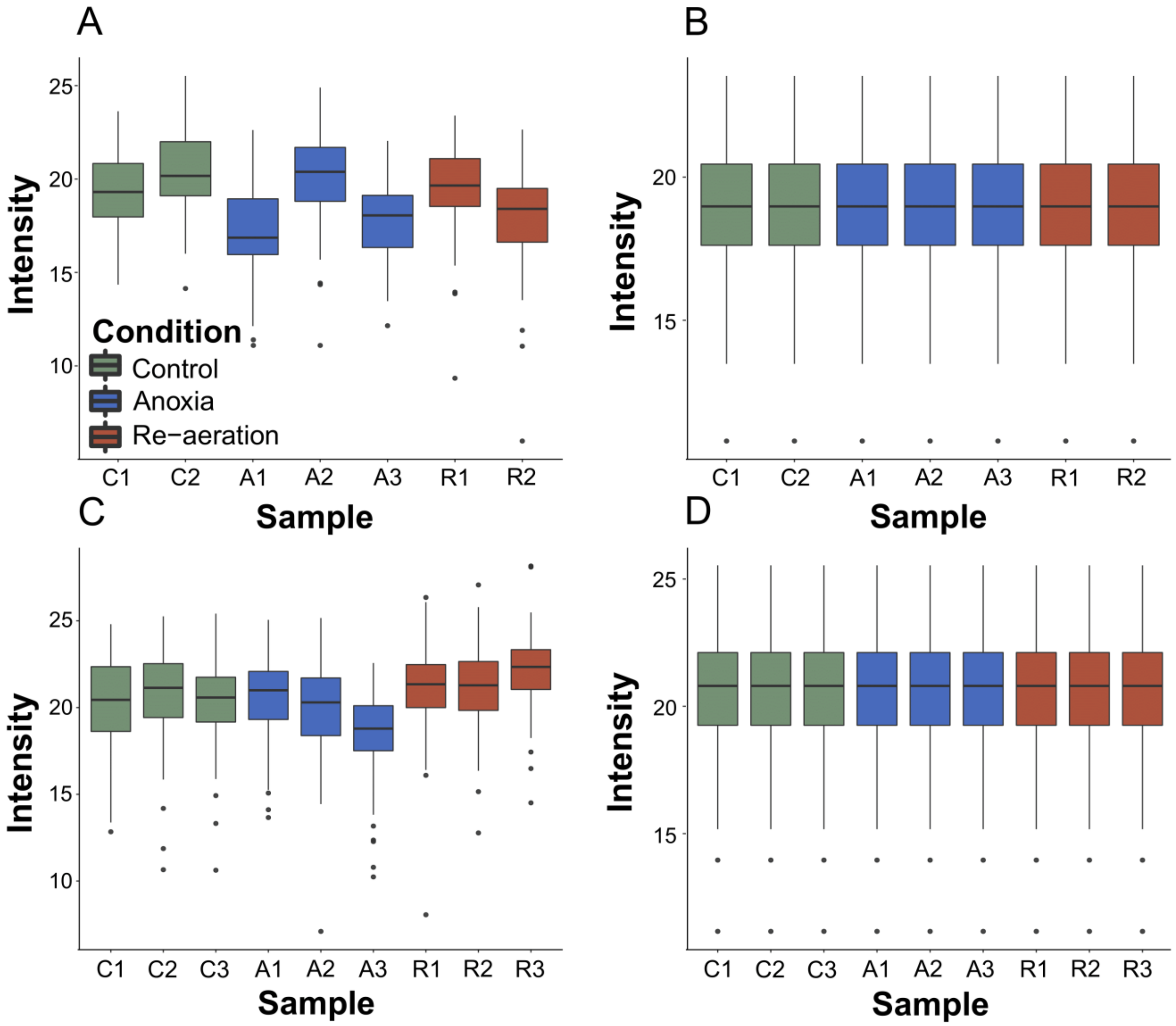

Supplementary Figure 2. Raw log-transformed (**A**) and normalized (**B**) mean intensities of the protein spots of shoot proteomes under control and experimental conditions. The (**C**) and (**D**) represent the same data for root proteomes. The intensities are based on pixel intensities within the spots calculated with the PDQuest software. The final normalized estimates are presented in **Supplementary Tables 9, 10**.

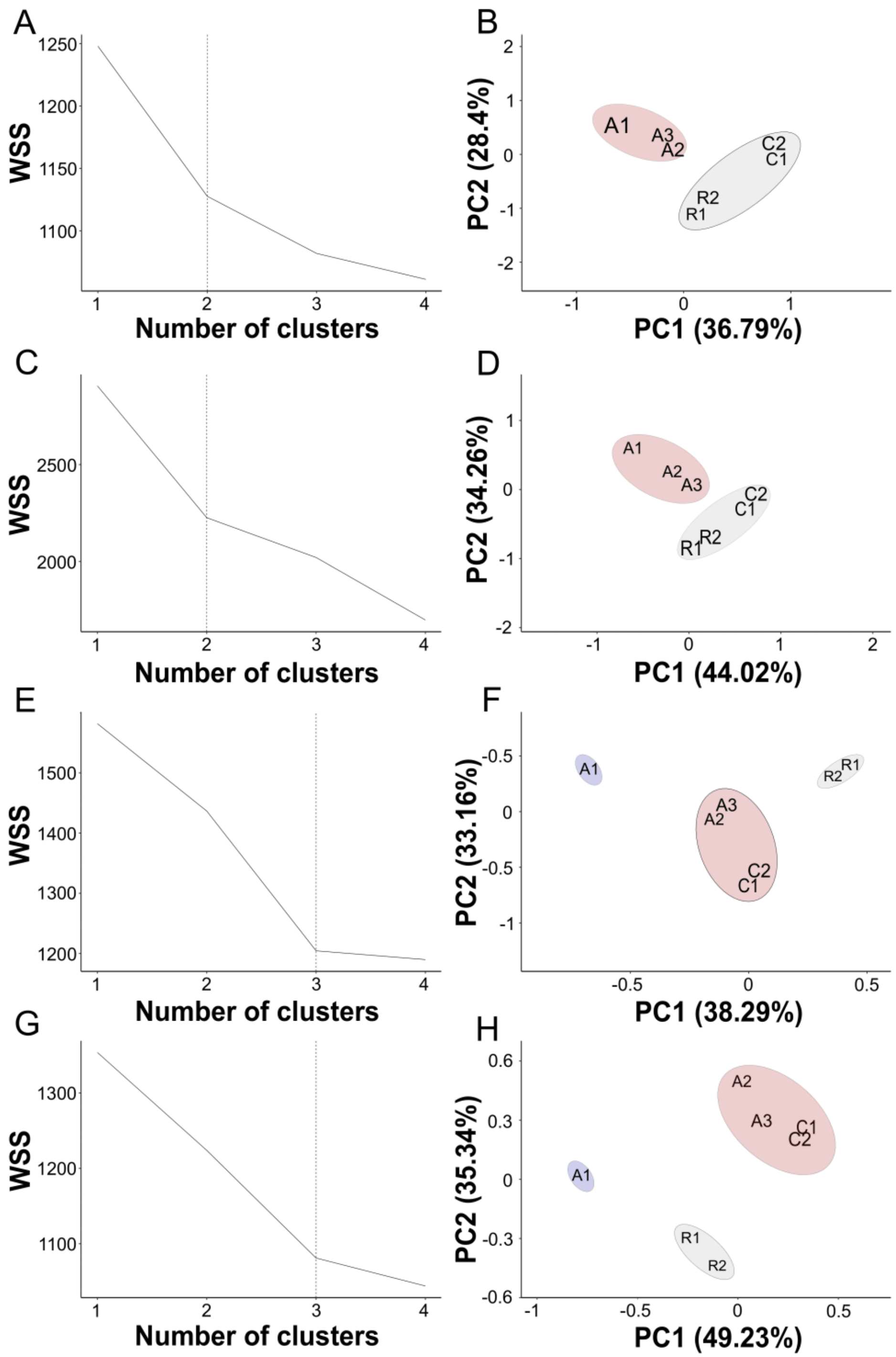

Supplementary Figure 3. The optimal number of clusters according to the elbow method and results of the k-means clustering procedure based on spots corresponding to four groups from shoot proteomes – all spots (**A, B**) significantly different spots (**C, D**), protein spots with annotations (**E, F**), significantly different protein spots proteins identified with mass spectrometry (**G, H**). The dashed lines on the plots on the left side of the panel indicate the optimal number of clusters based on the with-in-Sum-of-Squares (WSS) values. Capital letters denote experimental conditions: C – control, A – anoxia, and R – re-aeration.

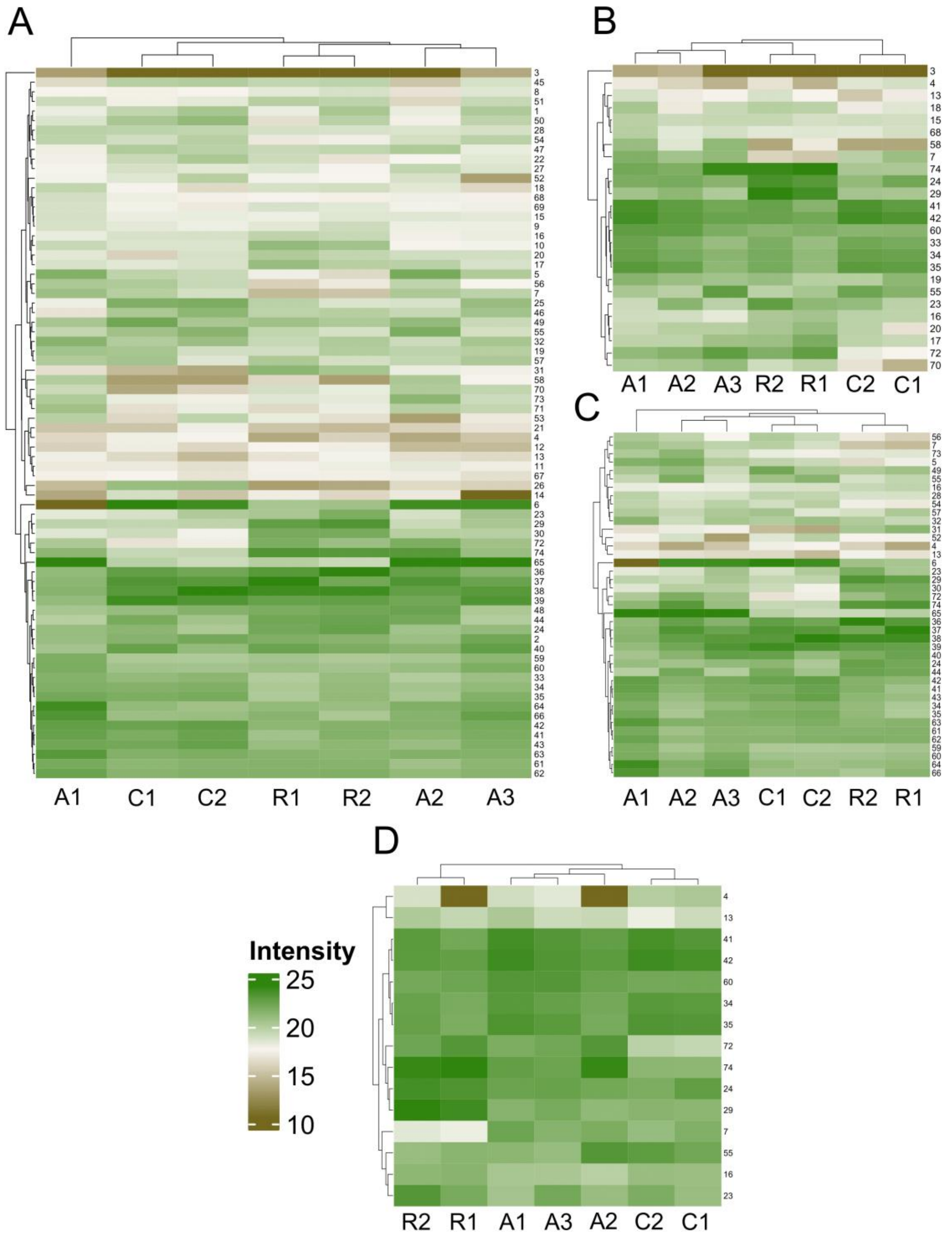

Supplementary Figure 4. The results of the hierarchical clustering procedure based on spots corresponding to four groups from shoot proteomes, namely, all spots (**A**), significantly different spots (**B**), protein spots with annotations (**C**), and significantly different protein spots with identified proteins (**D**). The letters correspond to experimental conditions: C – control, A – anoxia, and R – re-aeration. The rightmost numbers adjacent to heatmaps represent protein spots as shown in **Figure 2A**. Log-transformed and normalized intensities are based on pixel intensities within the spots calculated with the PDQuest software. The underlying raw data is provided in **Supplementary Table 9**.

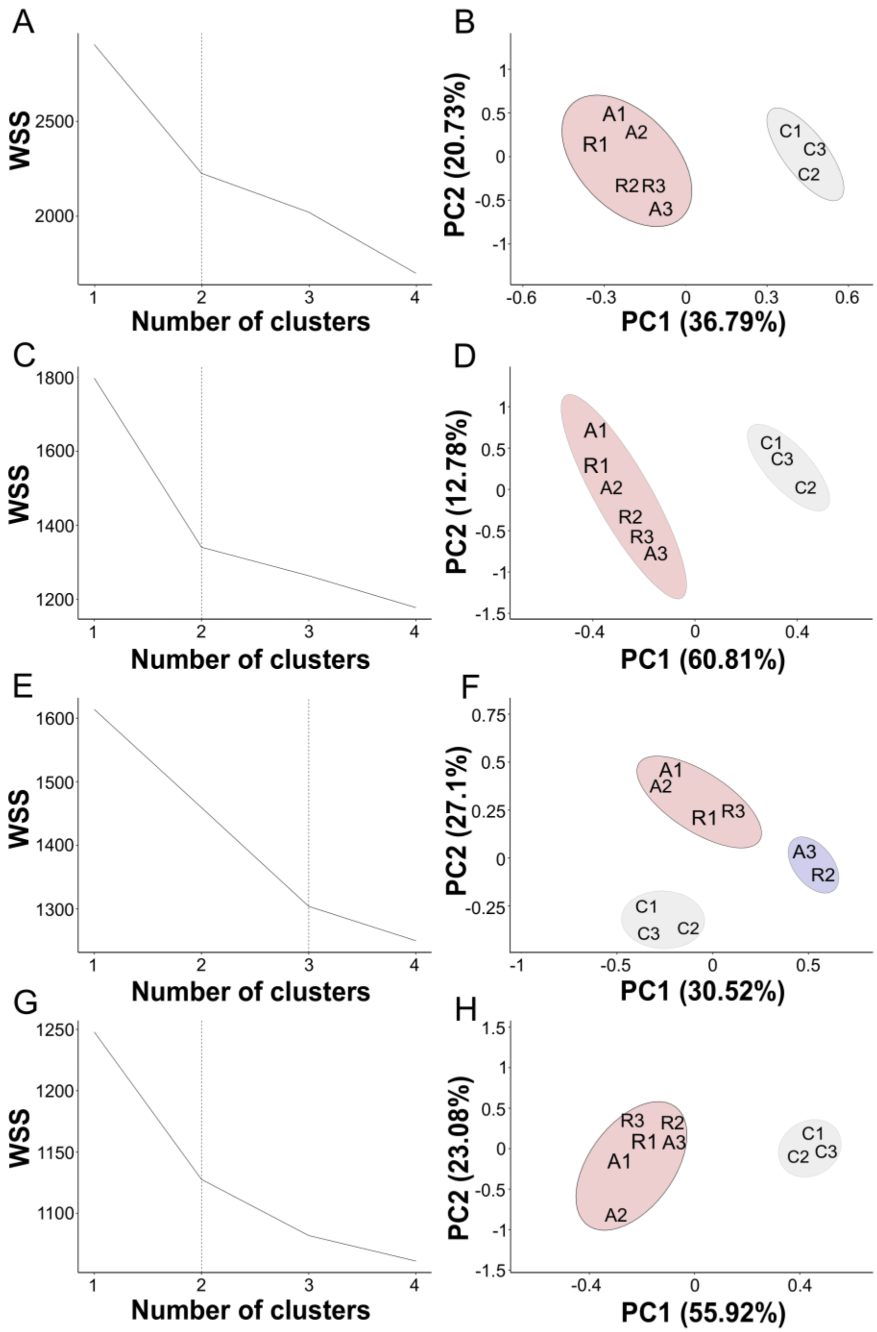

Supplementary Figure 5. The optimal number of clusters according to the elbow method and results of the k-means clustering procedure based on spots corresponding to four groups from root proteomes – all spots (**A, B**), significantly different spots (**C, D**), protein spots with annotations (**E, F**), and significantly different protein spots with proteins identified with mass spectrometry (**G, H**). The dashed lines on the plots on the left side of the panel indicate the optimal number of clusters based on the with-in-Sum-of-Squares (WSS) values. Experimental conditions are encoded with letters: C – control, A – anoxia, and R – re-aeration.

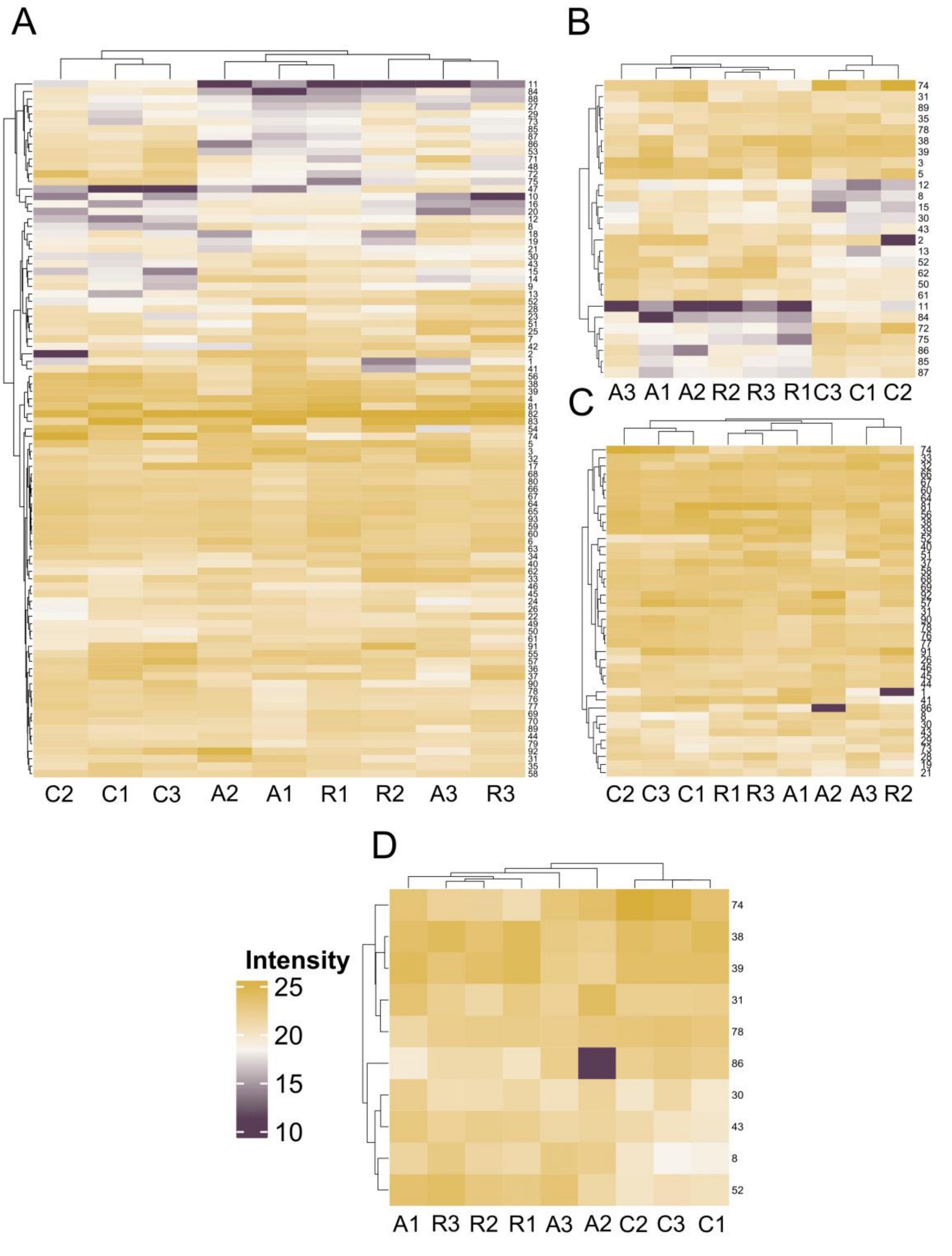

Supplementary Figure 6. The results of the hierarchical clustering procedure based on spots corresponding to four groups from root proteomes: all spots (**A**), significantly different spots (**B**), protein spots with annotations (**C**), and significantly different protein spots with identified proteins (**D**). Different letters denote experimental conditions: C – control, A – anoxia, and R – re-aeration. The numbers on the right side are protein spots presented in **Figure 2B**. Log-transformed and normalized intensity estimates are based on pixel intensities within the spots calculated with the PDQuest software. The underlying data with intensities is available in **Supplementary Table 10**.

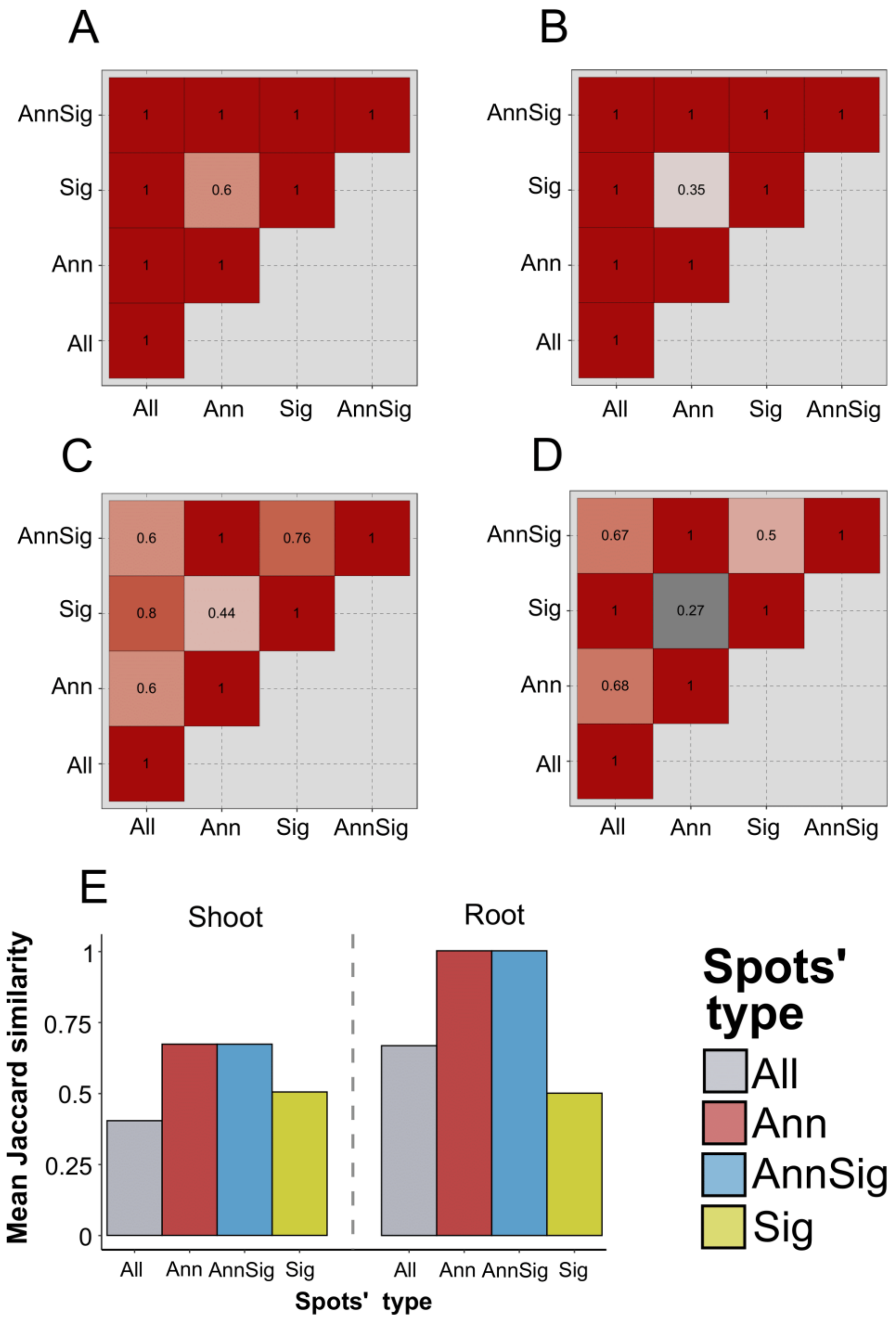

Supplementary Figure 7. The similarity between clusterizations of individual spots based on four groups from shoot (**A**) and root (**B**) proteomes – all spots (All), significantly different spots (Sig), protein spots with annotations (Ann), and significantly different protein spots with annotations (AnnSig) using the k-means clustering procedure. (**C**) Same similarity comparisons when utilizing hierarchical clustering procedures of the shoot and root (**D**) proteomes. (**E**) The similarity between k-means and hierarchical clusterization within the four above-described protein groups of shoot and root (**F**) proteomes.

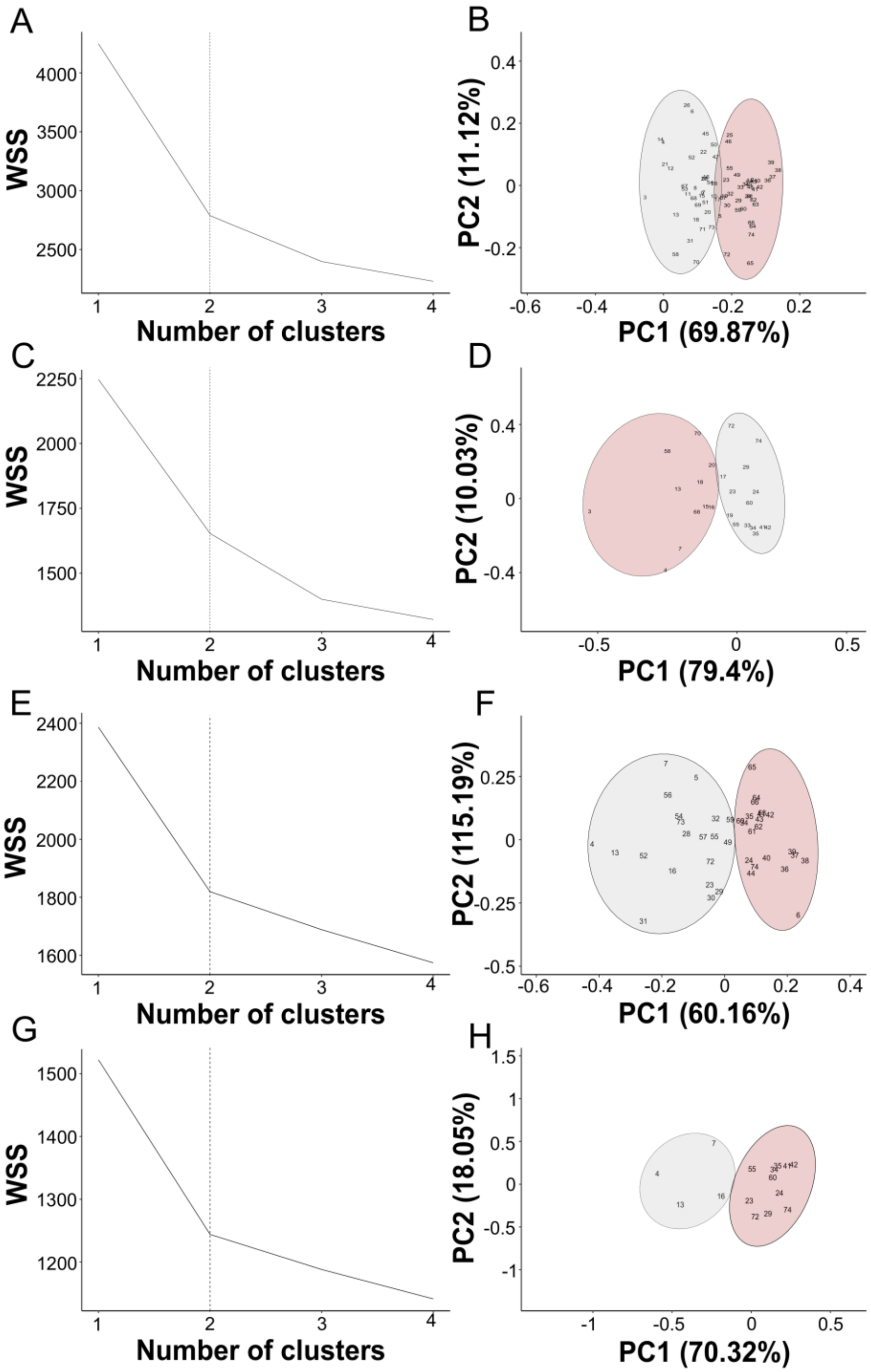

Supplementary Figure 8. The optimal number of clusters according to the elbow method and results of the k-means clustering procedure of individual spots corresponding to four groups from shoot proteomes – all spots (**A, B**) significantly different spots (**C, D**), protein spots with annotations (**E, F**), and significantly different protein spots with proteins identified with mass spectrometry (**G, H**). The dashed lines on the plots on the left side of the panel indicate the optimal number of clusters based on the with-in-Sum-of-Squares (WSS) values. The numbers inside the clusters represent protein spots according to **Figure 2A**. To see the spot-wise clustering patterns, consult **Supplementary Table 12**.

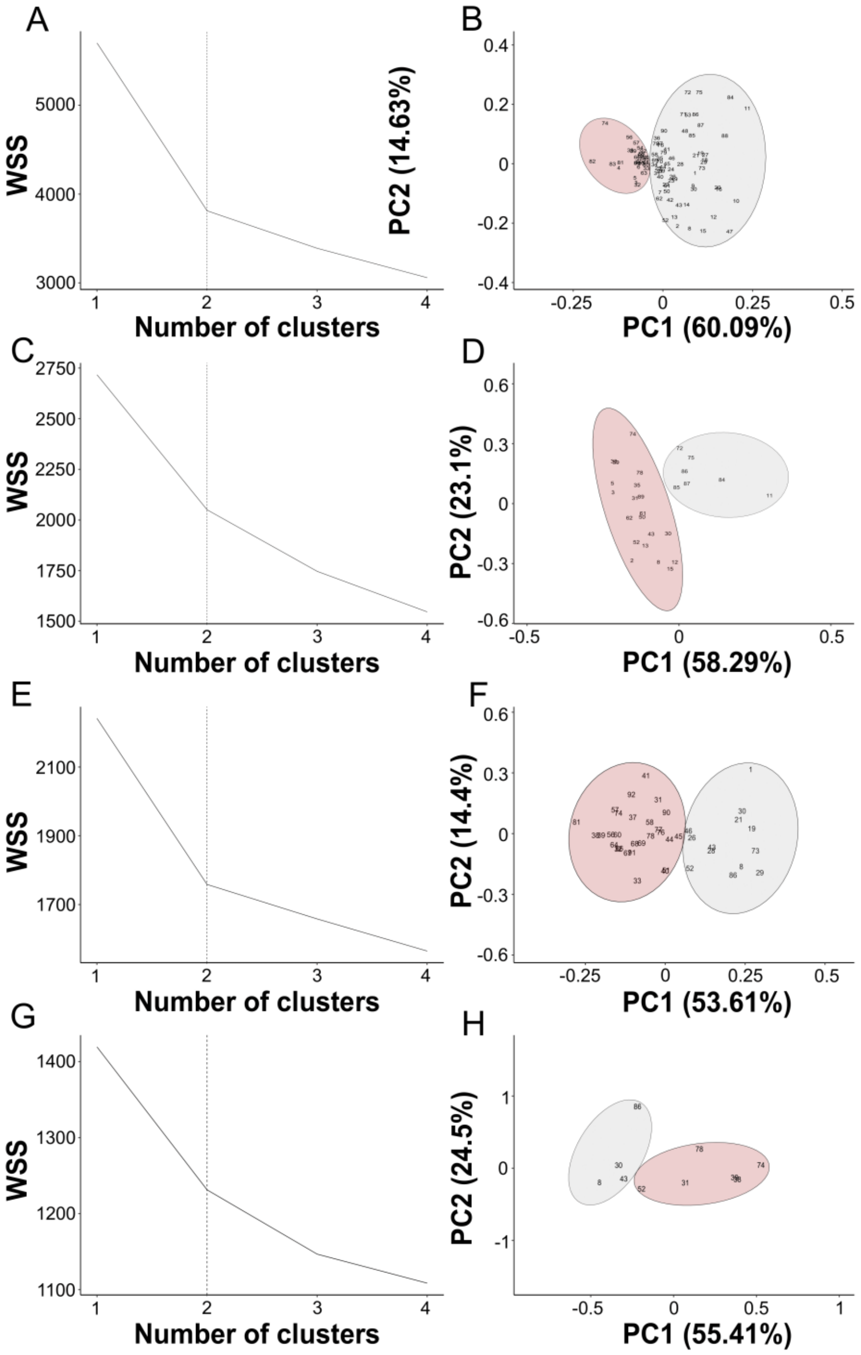

Supplementary Figure 9. The optimal number of clusters according to the elbow method and results of the k-means clustering procedure of individual spots corresponding to four groups from root proteomes – all spots (**A, B**) significantly different spots (**C, D**), protein spots with annotations (**E, F**), and significantly different protein spots with annotations (**G, H**). The dashed lines on the plots on the left side of the panel indicate the optimal number of clusters based on the with-in-Sum-of-Squares (WSS) values. The numbers inside the clusters correspond to protein spots as shown in **Figure 2B**. The spots' attributions according to clusters are presented in **Supplementary Table 12**.

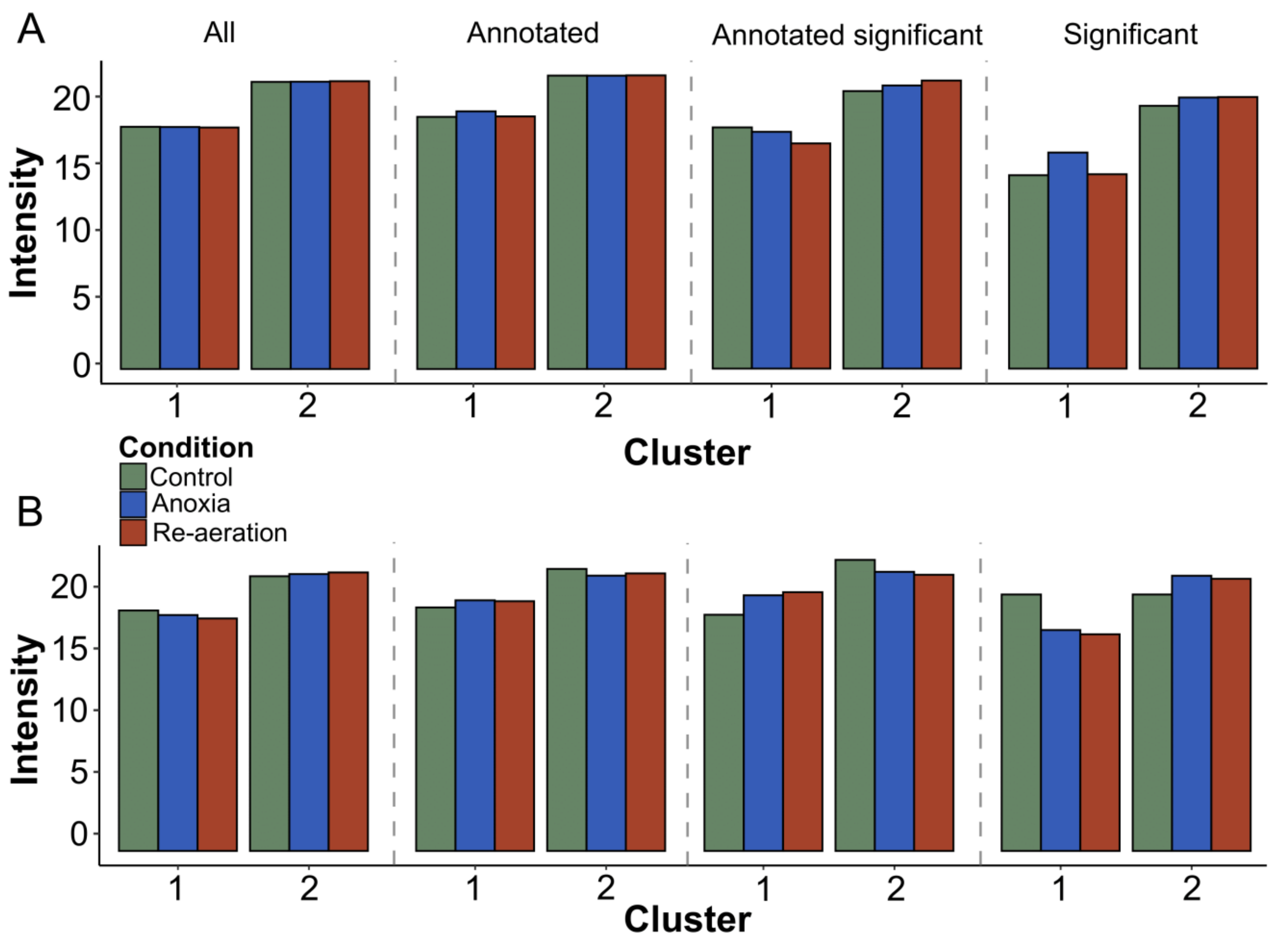

Supplementary Figure 10. Mean intensities of spots within clusters obtained using the k-means clustering procedure of individual spots corresponding to four groups from the shoot (**A**) and root (**B**) proteomes – all spots, significantly different spots, protein spots with annotations, and significantly different protein spots with proteins identified by mass spectrometry. Log-transformed and normalized intensities are based on pixel intensities within the spots calculated with the PDQuest software. The spots-to-clusters assignments and the respective intensities are available in **Supplementary Table 12**.

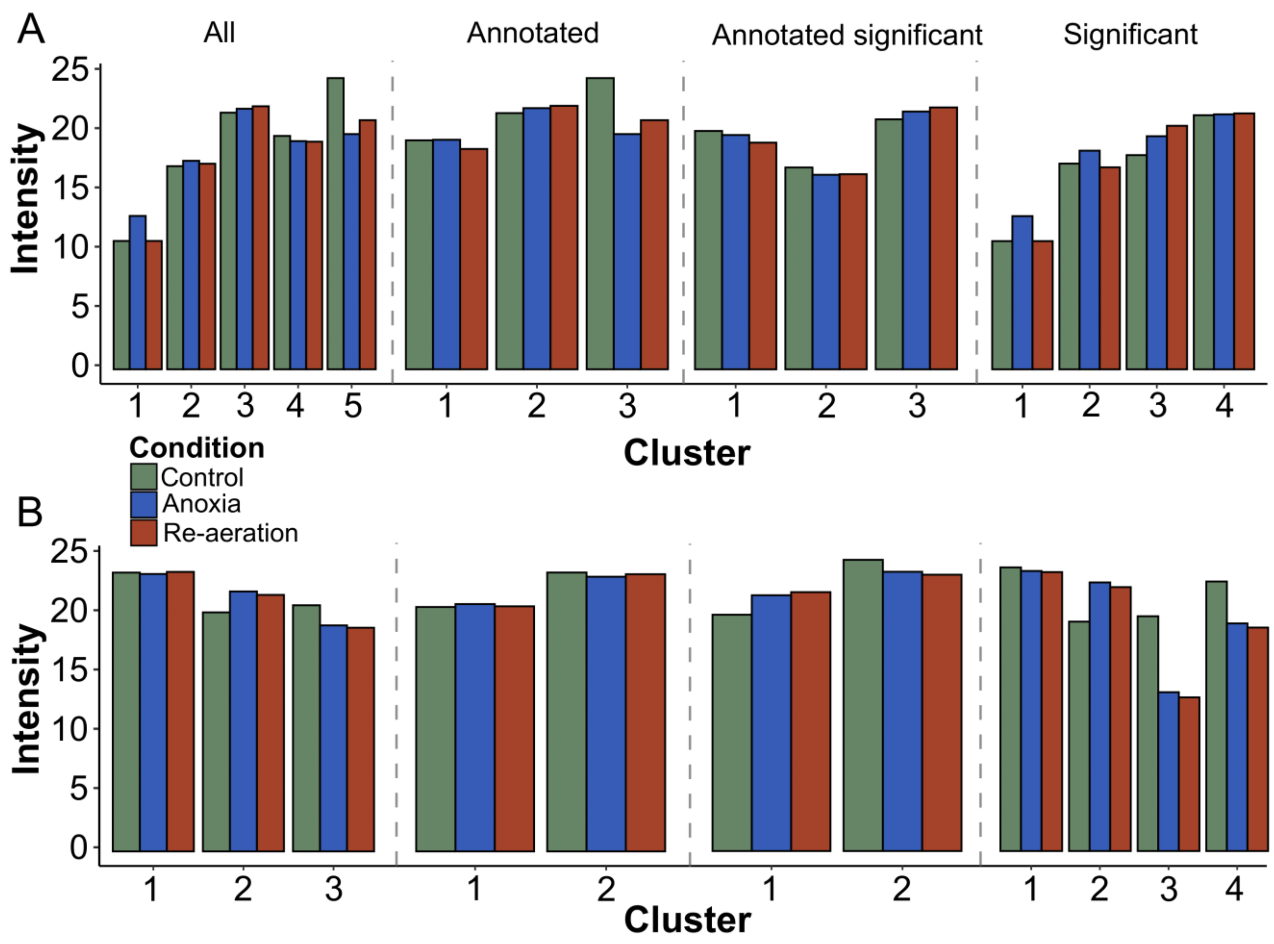

Supplementary Figure 11. Mean intensities of spots within clusters obtained using the hierarchical clustering procedure of individual spots corresponding to four groups from shoot (**A**) and root (**B**) proteomes – all spots, significantly different spots, protein spots with annotations, and significantly different protein spots with identified proteins. Log-transformed and normalized intensities are based on pixel intensities within the spots calculated with the PDQuest software. See **Supplementary Table 12** for cluster attributions coupled with spots' intensities.

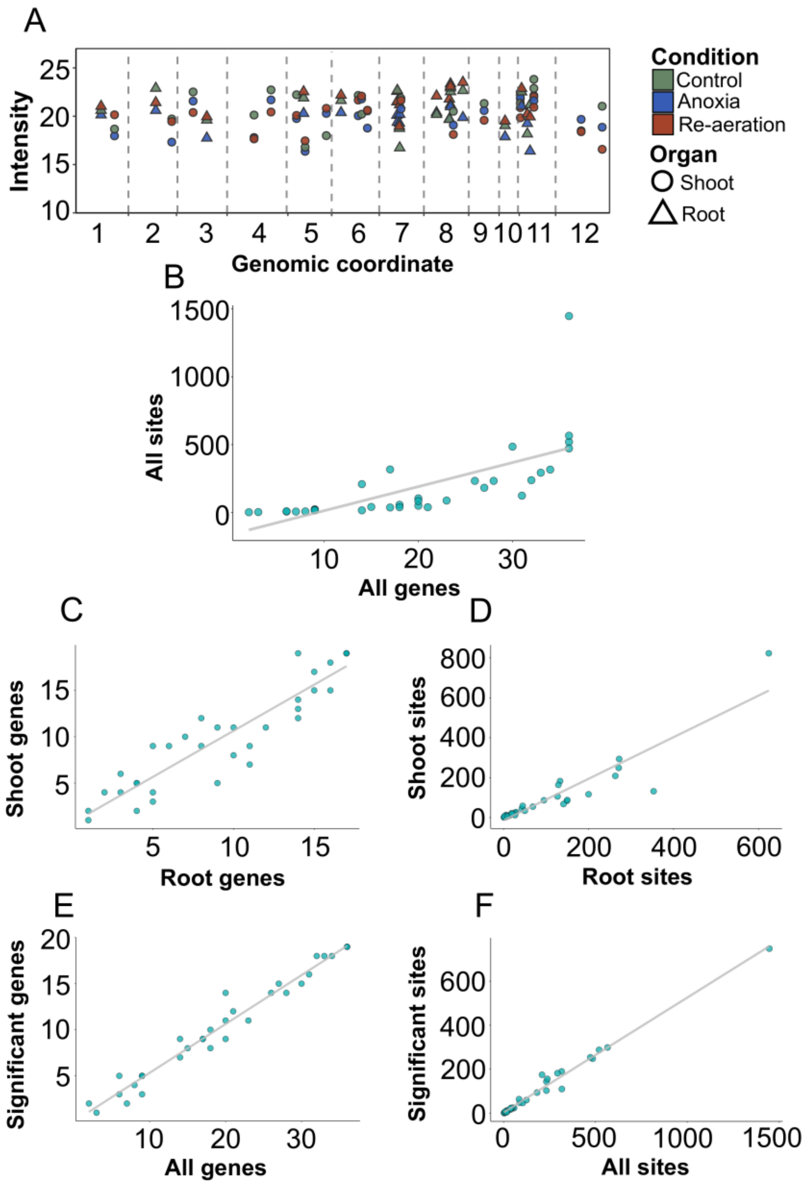

Supplementary Figure 12. The distribution of genes encoding proteins identified with mass spectrometry of root and shoot proteomes on the rice genome in relative genomic coordinates (b.p.) arranged following the numerical order of the chromosomes (**A**). Genes encoding shoot proteins are represented by circles and root ones – triangles. The mean intensities corresponding to different experimental conditions for spots corresponding to each protein are also presented. Log-transformed and normalized intensities are based on pixel intensities within the spots calculated with the PDQuest software. The exact locations of genomic loci, as well as accession numbers of proteins/genes, are presented in **Supplementary Tables 15, 16**, while intensities – in **Supplementary Table 13**. (**B**). The correspondence between the number of genes with predicted binding sites for a particular TF and the total number of sites for these TFs. (**C**) The relationship between the number of genes and sites with predicted binding sites based on all genes encoding all identified proteins and only significantly different proteins from shoots and roots (**D**). (**E**) The correspondence between the number of genes and sites with predicted binding sites based on all genes encoding identified proteins of the shoot and root (**F**) proteomes.

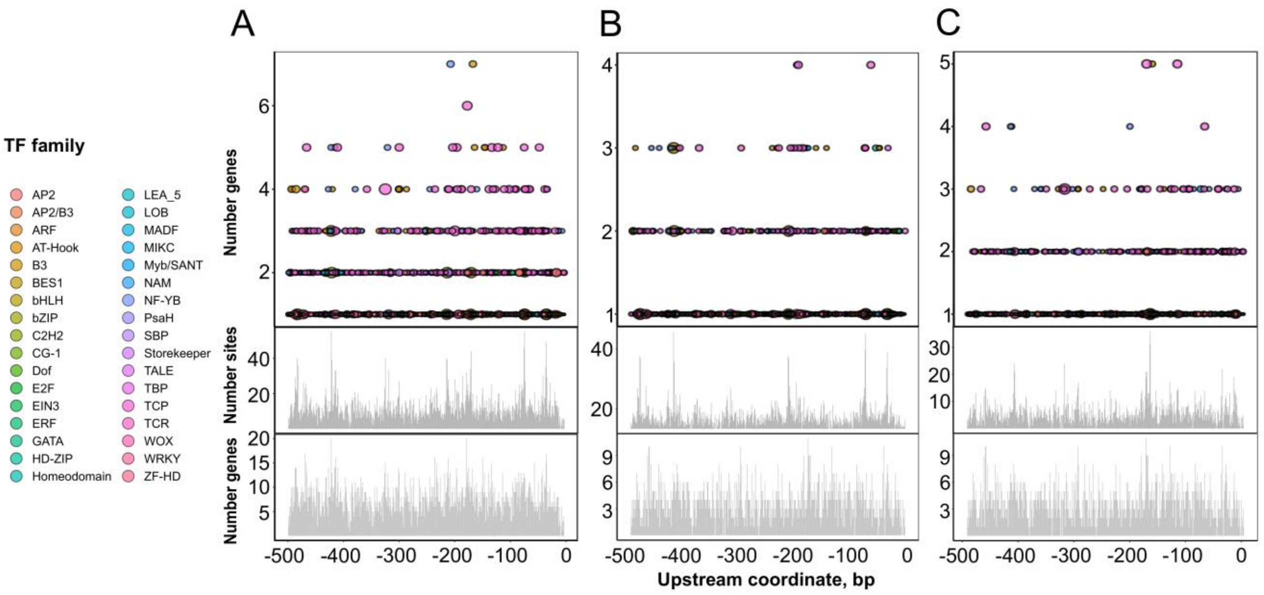

Supplementary Figure 13. The number of signals with predicted transcription factor binding sites in upstream regions of genes encoding all proteins from all spots (**A**), shoot (**B**), and root (**C**) proteomes. The upper panel shows the site-wise number of sites for a particular TF family. The middle panel demonstrates the total number of binding sites for a certain coordinate. The lower panel shows the number of genes in which a site of any TF family is detected.

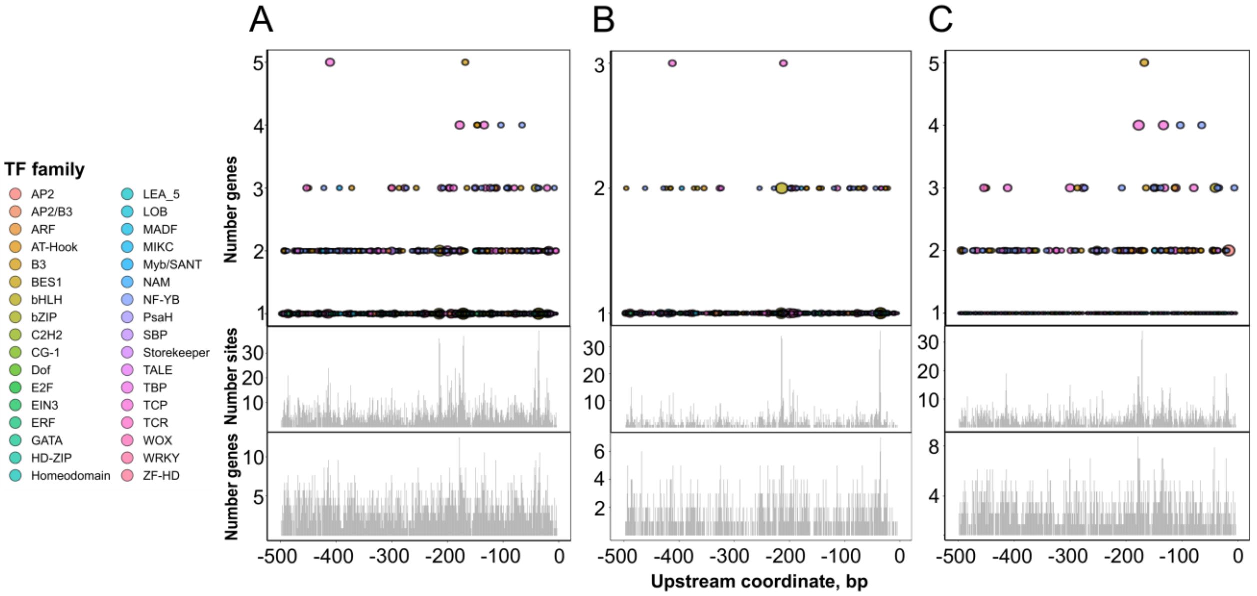

Supplementary Figure 14. The number of signals with predicted transcription factor binding sites in upstream regions of genes encoding significantly different proteins from all spots (**A**), shoot (**B**), and root (**C**) proteomes. The upper panel shows the site-wise number of sites for a particular TF family. The middle panel demonstrates the total number of binding sites for a certain coordinate. The lower panel shows the number of genes in which a site of any TF family is detected.

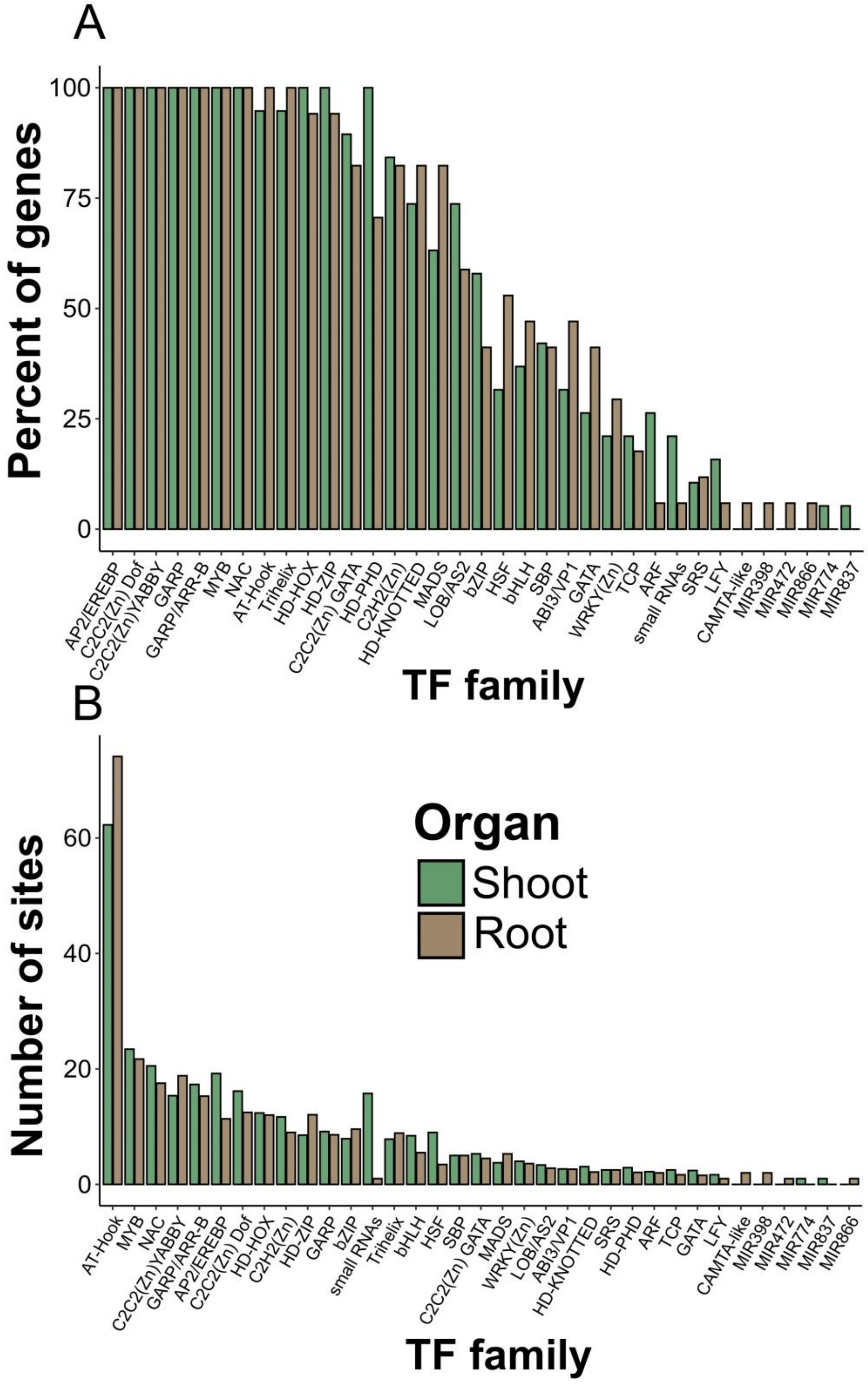

Supplementary Figure 15. (**A**) The percentage of genes having at least one predicted binding site of a particular TF family encoding *Arabidopsis thaliana* orthologs of identified proteins from shoots and roots. (**B**) The mean number of predicted binding sites of a particular TF family per respective gene. Accession numbers of *A. thaliana* homologs found using the Ensembl resource are available in **Supplementary Tables 15, 16**.

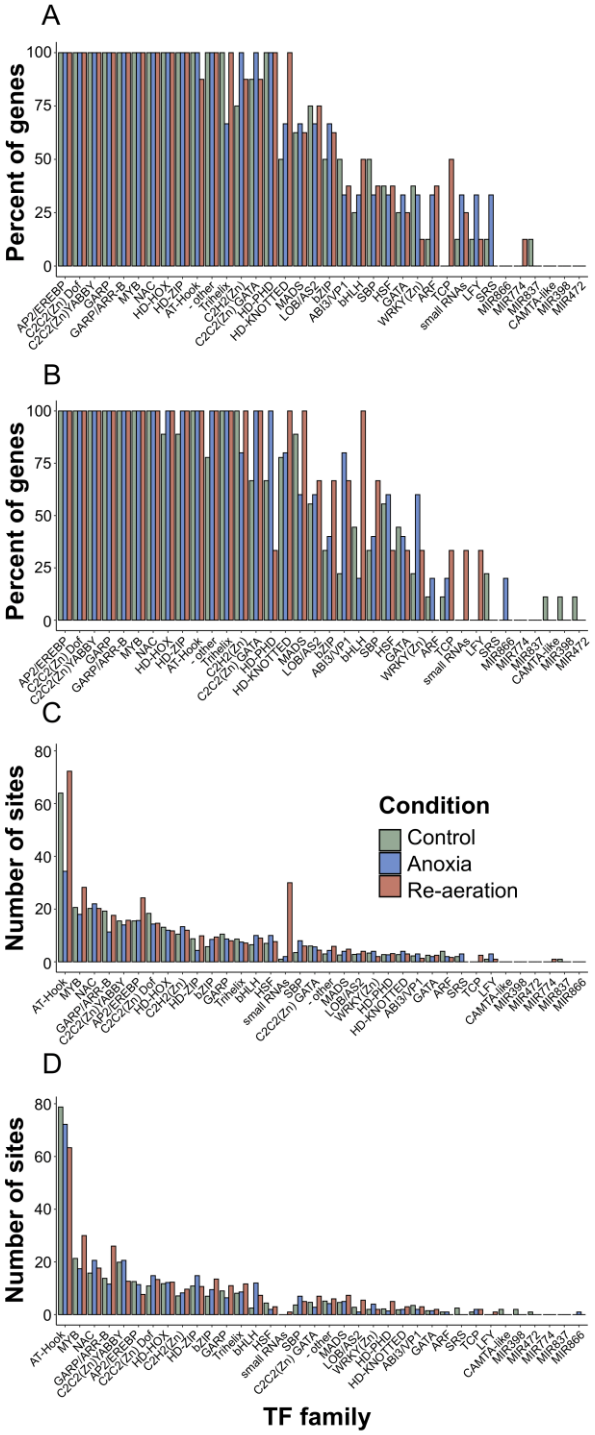

Supplementary Figure 16. (**A**) The percent of genes encoding *Arabidopsis thaliana* orthologs of identified proteins from shoots with at least one TFBS of a certain TF family, grouped according to the condition in which the intensity of the respective spots was the highest. (**C**) The same for root proteins. (**B**) The mean number of sites of a certain TF family in the above-mentioned genes from shoots grouped attributed to a certain condition as described above. (**D**) The same for root proteins. To see condition- and organ-wise estimates, consult **Supplementary Table 20**.

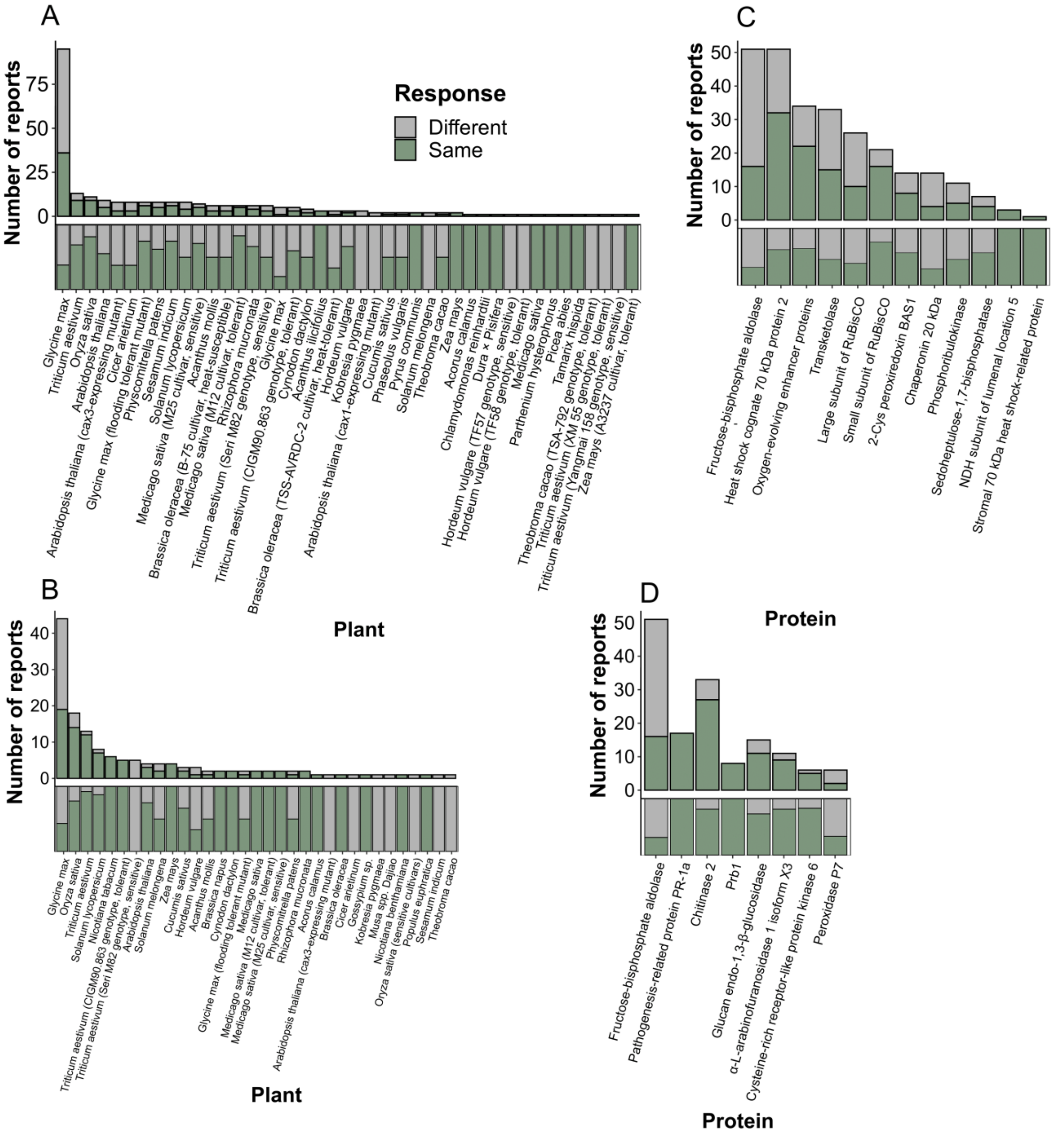

Supplementary Figure 17. Comparisons between plant responses to biotic and abiotic stress in terms of protein abundance of significantly different annotated proteins identified in rice shoot (**A**) and root (**B**) proteomes. (**C**) The same comparisons for individual proteins were identified in rice shoots and roots (**D**). The color indicates the percentage of reports (the effect of a certain stressor in a particular research item) in which the protein shows the same behavior (increases or decreases in the respective conditions) as reported in the current study. In the case of plants, a summary of all proteins is given. The bottom adjacent plots display the overall percentage of similar/unequal plant responses to stress. The data used for generating plots is available in **Supplementary Tables 22, 23**.

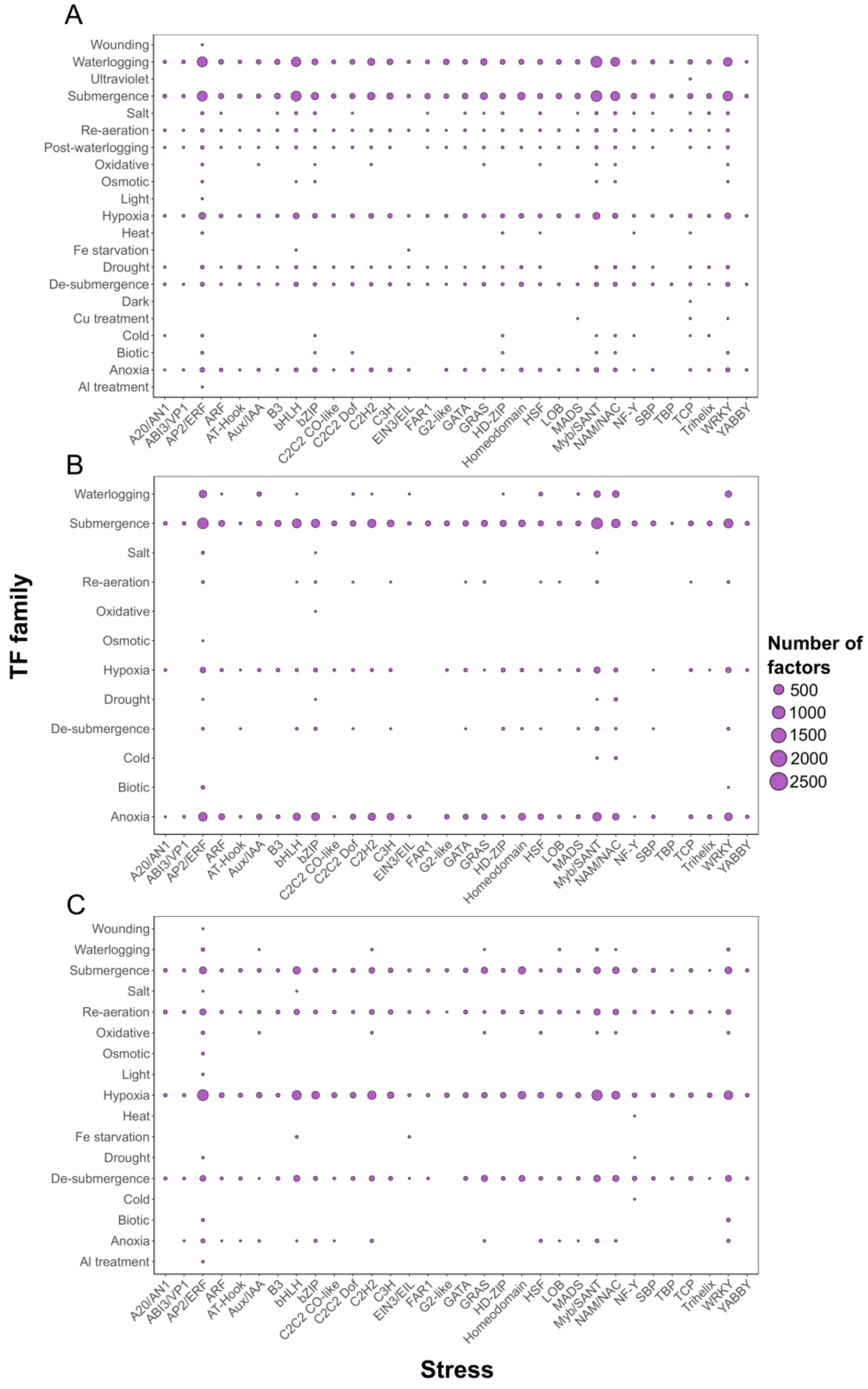

Supplementary Figure 18. The distribution of transcription factors attributed to families during exposures of various plants (**A**), rice (**B**), and *A. thaliana* (**C**). The size of the dots is proportional to the number of transcription factors detected in the studies, which are presented in **Supplementary Table 26**. Shown are all TFs affected, i.e., down-regulated and up-regulated moieties are counted jointly.

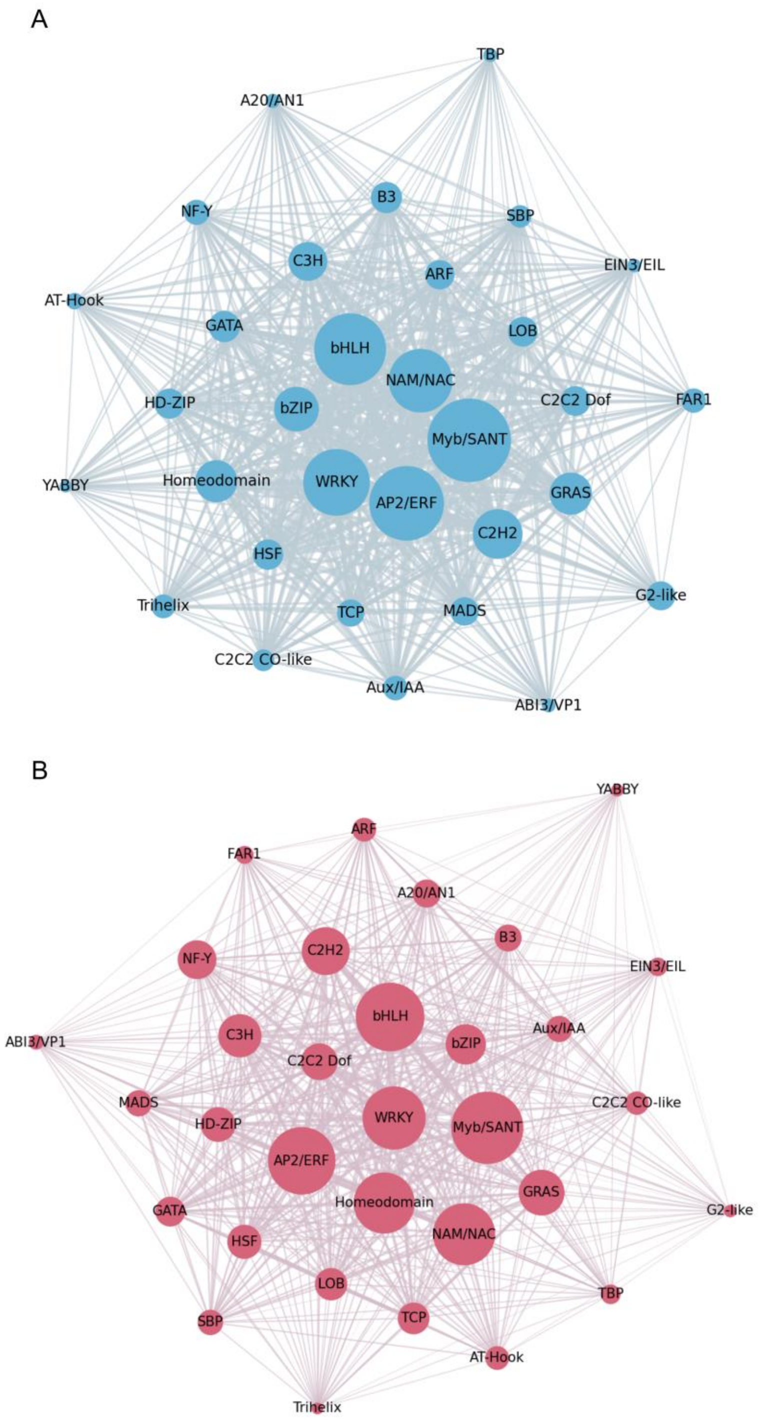

Supplementary Figure 19. The co-occurrence graph of TF families affected during oxygen depletion (**A**) and reoxygenation (**B**). Nodes represent TF groups, whilst the edges link families in case representatives of the family were detected together, with the size and the weight being proportional to the number of individual TFs and reports, respectively. The underlying data is available in **Supplementary Table 26**. Shown are all TFs affected, i.e., down-regulated and up-regulated moieties are counted jointly.
